# A Compact Model for the Complex Plant Circadian Clock

**DOI:** 10.3389/fpls.2016.00074

**Published:** 2016-02-05

**Authors:** Joëlle De Caluwé, Qiying Xiao, Christian Hermans, Nathalie Verbruggen, Jean-Christophe Leloup, Didier Gonze

**Affiliations:** ^1^Unité de Chronobiologie Théorique, Faculté des Sciences, Université Libre de BruxellesBrussels, Belgium; ^2^Laboratory of Plant Physiology and Molecular Genetics, Faculté des Sciences, Université Libre de BruxellesBrussels, Belgium

**Keywords:** circadian rhythms, *Arabidopsis thaliana*, mathematical model, entrainment by light–dark cycles, clock mutants

## Abstract

The circadian clock is an endogenous timekeeper that allows organisms to anticipate and adapt to the daily variations of their environment. The plant clock is an intricate network of interlocked feedback loops, in which transcription factors regulate each other to generate oscillations with expression peaks at specific times of the day. Over the last decade, mathematical modeling approaches have been used to understand the inner workings of the clock in the model plant *Arabidopsis thaliana*. Those efforts have produced a number of models of ever increasing complexity. Here, we present an alternative model that combines a low number of equations and parameters, similar to the very earliest models, with the complex network structure found in more recent ones. This simple model describes the temporal evolution of the abundance of eight clock gene mRNA/protein and captures key features of the clock on a qualitative level, namely the entrained and free-running behaviors of the wild type clock, as well as the defects found in knockout mutants (such as altered free-running periods, lack of entrainment, or changes in the expression of other clock genes). Additionally, our model produces complex responses to various light cues, such as extreme photoperiods and non-24 h environmental cycles, and can describe the control of hypocotyl growth by the clock. Our model constitutes a useful tool to probe dynamical properties of the core clock as well as clock-dependent processes.

## 1. Introduction

The circadian clock is an endogenous timekeeper that allows organisms to anticipate the day/night cycle, thus improving their fitness (Green et al., 2002; Dodd et al., 2005). In plants, the clock has been shown to regulate a wide variety of processes (Barak et al., 2000), including hypocotyl and root growth (Yazdanbakhsh et al., 2011), flowering time (Imaizumi et al., 2005; Johansson and Staiger, 2015), sugar metabolism (Dodd et al., 2015), photosynthesis (Haydon et al., 2013; Webb and Satake, 2015), nutrient homeostasis (Haydon et al., 2015), hormonal signaling, and immunity (Bolouri Moghaddam and Van den Ende, 2013). Circadian oscillations originate at the cellular level from the interactions of at least a dozen clock genes (Nagel and Kay, 2012). Because the circadian network is made up of many interconnected feedback loops, its behavior is hard to grasp by sheer intuition, and mathematical modeling is a very useful tool to help understand it.

The first conceptual models of the oscillator in the model plant *Arabidopsis thaliana* were put forward shortly after the identification of the clock genes *CIRCADIAN CLOCK ASSOCIATED 1 (CCA1), LATE ELONGATED HYPOCOTYL (LHY)* and *TIMING OF CAB EXPRESSION 1 (TOC1)*. For example, Alabadí et al. (2001) proposed a positive-negative feedback loop between CCA1/LHY and TOC1, based on the experimentally observed mutual regulation, while Matsushika et al. (2000) hypothesized a “bar code clock” made up of the five members of the PSEUDO-RESPONSE REGULATOR (PRR) gene family, which are expressed sequentially through the day. Locke et al. (2005a) built the very first mathematical model, using ordinary differential equations (ODEs) to describe the temporal evolution of the concentration of various clock components. This first model was a formal description of the previously mentioned feedback loop between CCA1, LHY, and TOC1, in which the two partially redundant morning-phased genes *CCA1* and *LHY* inhibit the expression of evening-phased *TOC1*, which activates them in turn. This simple mechanism was sufficient to generate a 24 h free-running rhythm, but could not reproduce more complex features such as photoperiodic adaptation or mutant phenotypes. It was nevertheless a useful first step, and in the following decade, as more clock components were discovered, further extensions of the Locke model were published (Locke et al., 2005b, 2006; Zeilinger et al., 2006; Pokhilko et al., 2010, 2012, 2013; Fogelmark and Troein, 2014). Each iteration built upon the previous one, adding new genes and regulations, and occasionally revising the role of a component as new evidence was made available. The most important revision concerns the role of the transcription factor TOC1: initially thought to be an activator of *CCA1/LHY*, it was later shown to be an inhibitor of many clock components, including *CCA1/LHY* (Gendron et al., 2012).

Other approaches that are not based on ODEs were also developed, such as the Boolean model described in Akman et al. (2012). Linear time invariant (LTI) systems, a type of black-box model, have successfully been used to investigate the response of the clock to environmental stimuli (Dalchau, 2012). Each type of model has its own strengths and weaknesses, and different approaches can fulfill different roles in the study of the circadian clock. For example, LTI systems have accurately predicted which clock gene senses a particular environmental signal, but do not offer information about the mechanism driving the oscillations.

A more recent trend is the study and modeling of *Ostreococcus tauri*, a unicellular alga whose core circadian clock is made up of a single loop between orthologs of CCA1 and TOC1 (Corellou et al., 2009). This system has been modeled and used to study light sensing and entrainment (Troein et al., 2011; Dixon et al., 2014; Thommen et al., 2015).

In this work, we present an ODE-based model for *A. thaliana* that retains the small number of components found in older models of the same type, but incorporates many more recent discoveries into the wiring of the oscillator. Compared to the latest published models (Pokhilko et al., 2013; Fogelmark and Troein, 2014), our minimal model is more qualitative. However, its small size makes it more tractable. We show that the many connections between the model components and the addition of multiple light inputs are sufficient to reproduce a large range of behaviors in various light conditions. We also demonstrate the ability of our model to describe clock-dependent processes by building a small output module that reproduces hypocotyl growth phenotypes.

## 2. Model

### 2.1. The model includes four pairs of genes

**Figure 1:**
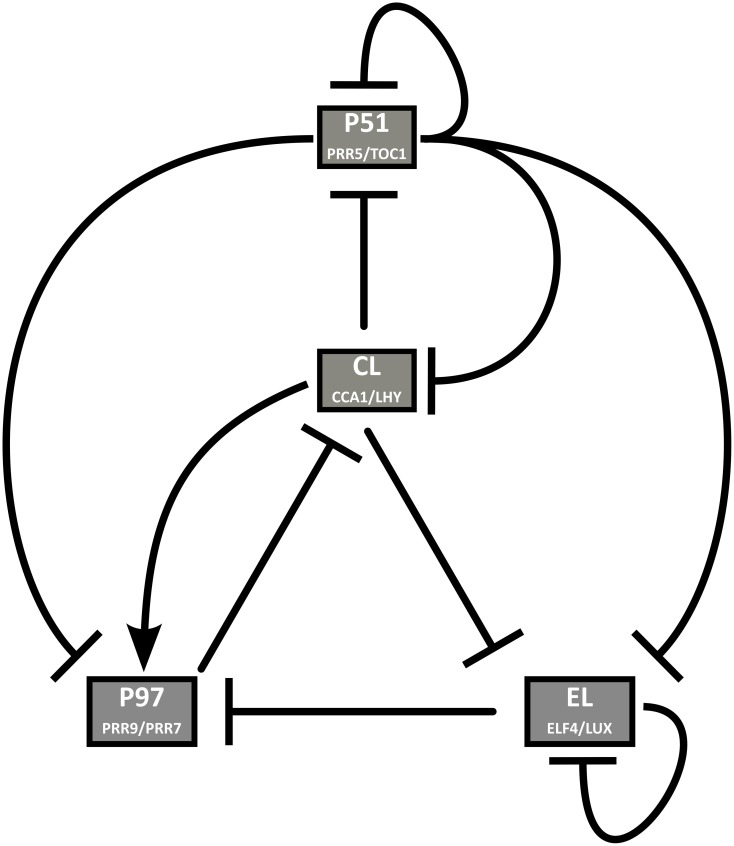
**Clock model structure**. CL represents the dawn genes *CCA1* and *LHY*, P97 represents the morning genes *PRR9* and *PRR7*, P51 represents the evening genes *PRR5* and *TOC1*, and EL represents *ELF4* and *LUX*.

Given the large number of known clock components and the high degree of redundancy between some of them, we chose to merge very similar genes into single variables, which allowed us to include many well-characterized genes without needing several sets of almost identical equations. This approach is not novel, and has successfully been used with several clock genes, in particular *CCA1* and *LHY*, which were treated as a single entity in all previous ODE-based models except in Fogelmark and Troein (2014). In Locke et al. (2006), Zeilinger et al. (2006), and Pokhilko et al. (2010), *PSEUDO-RESPONSE REGULATOR9 (PRR9)*, and *PSEUDO-RESPONSE REGULATOR7 (PRR7)* were also combined into a single variable. Here, we extended the same approach to two more pairs of genes. Our model structure, shown in Figure 1, includes four groups of two genes each. Each pair of genes has similar expression profiles, regulators, targets, and defects in loss-of-function mutant lines.

#### 2.1.1. Dawn genes: *CCA1* and *LHY*

The dawn-phased genes *CCA1* and *LHY* are transcription factors belonging to the MYB superfamily (Schaffer et al., 1998; Wang and Tobin, 1998). They play largely, although not completely, redundant roles within the clock (Mizoguchi et al., 2002). They are negatively regulated by the *PRR* family of genes (Nakamichi et al., 2010), and have been shown to inhibit the expression of many evening-phased genes, including the clock components *TOC1* (Alabadí et al., 2001), *EARLY FLOWERING 4 (ELF4)* (Kikis et al., 2005), and *LUX ARRHYTHMO (LUX)* (Hazen et al., 2005). CCA1 and LHY also bind to the promoters of the morning genes *PRR9* and *PRR7*, and have a positive effect on their expression (Farré et al., 2005), although it is unclear whether that action is direct or indirect (Kawamura et al., 2008). The expression of both genes is induced by the dark to light transition, and light simultaneously promotes the translation and degradation of the *LHY* mRNA (Kim et al., 2003).

In our model, the expression of *CCA1/LHY* is transiently activated by light and inhibited by PRR9/PRR7 and PRR5/TOC1. The translation and mRNA degradation rates are increased by light. The protein inhibits the expression of the evening genes *ELF4/LUX* and *PSEUDO-RESPONSE REGULATOR (PRR5)/TOC1*, and directly promotes that of the morning genes *PRR9/PRR7*.

#### 2.1.2. Dusk genes: *ELF4* and *LUX*

The Evening Complex (EC) is made up of three proteins, *EARLY FLOWERING 3 (ELF3), ELF4* and *LUX* (Nusinow et al., 2011). Of the three, LUX is the only proper transcription factor (Onai and Ishiura, 2005; Helfer et al., 2011) but the formation of the EC is necessary for LUX to become active (Mizuno et al., 2014b). The EC acts as a transcriptional repressor that inhibits the expression of the morning genes *PRR9* and *PRR7*, its own component *LUX*, as well as the growth- and flowering-related genes *PHYTOCHROME INTERACTING FACTOR 4 (PIF4)* and *PHYTOCHROME INTERACTING FACTOR 5 (PIF5)* (Mizuno et al., 2014b). Two other clock genes, *ELF4* and *TOC1*, may also be targets, as suggested by the elevated levels of expression of those genes in mutants lacking the EC and the presence of at least one LUX binding site in their promoters. However, the EC components were not shown to bind to those sites *in vivo* (Onai and Ishiura, 2005; Dixon et al., 2011; Helfer et al., 2011; Mizuno et al., 2014b).

In light/dark cycles, *ELF4* and *LUX* have very similar expression profiles, with a peak at dusk or around 12 h after dawn, whichever is earlier (Doyle et al., 2002; Hazen et al., 2005), while the expression of *ELF3* extends much later into the night (Hicks et al., 2001). The expression of *ELF3* is only inhibited by CCA1/LHY, while *ELF4* and *LUX* are inhibited by CCA1/LHY, TOC1, and the EC. Additionally, *ELF4* is induced by light signaling-related proteins (Li et al., 2011).

In the model, we have assumed that ELF4 or LUX must be the limiting factor in the formation of the EC, and have not included ELF3 explicitly. One equation represents the mRNA of *ELF4/LUX*, and another the fully functional EC, which is translated directly from the mRNA without intermediate steps. The expression of *ELF4/LUX* is modeled as light dependent, and inhibited by CCA1/LHY, PRR5/TOC1, and the EC. The EC represses *PRR9/PRR7* and *ELF4/LUX* but not *PRR5/TOC1*, even though there is a possibility that this interaction exists. The experimental evidence in support of a direct inhibition of *PRR9, PRR7*, and *LUX* is strong, while the case for a similar regulation of *ELF4* and *TOC1* is slightly less clear cut, and nothing suggests an effect on *PRR5* (Mizuno et al., 2014b). Therefore, we based our decision mainly on *LUX* for the *ELF4/LUX* pair and *PRR5* for the *PRR5/TOC1* pair.

#### 2.1.3. The PRR family: *PRR9* and *PRR7, PRR5* and *TOC1*

The *A. thaliana PSEUDO-RESPONSE REGULATOR* family contains five clock-related genes: *PRR9, PRR7, PRR5, PRR3*, and *TOC1* (sometimes known as *PRR1*) (Matsushika et al., 2000). We have excluded *PRR3* from our model because its expression is limited to vascular tissue (Para et al., 2007). Furthermore, this gene has not been characterized as extensively as the other four family members.

The PRRs are transcriptional repressors, acting to inhibit *CCA1* and *LHY* as well as each other (Nakamichi et al., 2010; Gendron et al., 2012; Huang et al., 2012). TOC1 also inhibits *ELF4* and *LUX* (Huang et al., 2012). While each *PRR* is partially redundant with the next one, there is little overlap between the expression, regulation and roles of the two most distant members, *PRR9* and *TOC1*. We chose to split the *PRR* family into two pairs, *PRR9/PRR7* and *PRR5/TOC1*, based on the transcriptional and post-translational regulation of all four genes and the clock-related defects in overexpression and loss-of-function mutant lines.

The circadian phenotypes of the various *prr* knockouts are one argument in favor of those pairings: *prr5, toc1* and *prr5toc1* mutants have a short period and lowered *CCA1/LHY* expression levels (Ito et al., 2008), while the *prr9, prr7*, and *prr9prr7* mutants have long periods and slightly elevated *CCA1/LHY* levels (Farré et al., 2005).

At the transcriptional level, *PRR9* and *PRR7* are activated by CCA1 and LHY (Farré et al., 2005), while *TOC1* is inhibited (Alabadí et al., 2001). No direct evidence is available regarding *PRR5* but several observations indicate its inhibition by CCA1 and LHY. Its circadian phase is similar to that of *TOC1, ELF4, LUX*, and *GIGANTEA (GI)*, all of which are known targets of CCA1 and LHY (Pokhilko et al., 2012). It is also activated by the MYB transcription factor REVEILLE8 (RVE8), which works in opposition to CCA1 and LHY at the molecular level, and which positively regulates all of the aforementioned evening-phased genes (Rawat et al., 2011; Hsu et al., 2013). It should be noted that *RVE8* is not explicitly included in our model, but its action is implicitly accounted for in the description of *CCA1/LHY*. Furthermore, *PRR9* and *PRR7* are inhibited by the evening complex in a temperature-dependent fashion. There is no evidence of the EC targeting *PRR5*, and *PRR5* and *TOC1* are not strongly temperature-responsive like *PRR9* and *PRR7* (Mizuno et al., 2014a,b).

At the post-translational level, all of the PRRs are degraded faster in the dark than in the light but the proteins mediating that effect are different: PRR5 and TOC1 are tagged for degradation by ZEITLUPE (ZTL) (Más et al., 2003; Kim et al., 2007; Fujiwara et al., 2008), which does not interact with PRR9 or PRR7 (Kiba et al., 2007; Kim et al., 2011). The protein acting on PRR9 and PRR7 has not been identified yet (Farré and Kay, 2007; Ito et al., 2007). Although neither ZTL nor the unknown regulator of PRR9/PRR7 is explicitly included in the model, this provides another indication that the two pairs *PRR9/PRR7* and *PRR5/TOC1* are more similar to each other than to any other *PRR*. Moreover, PRR5 and TOC1 form a dimer which enhances the nuclear import and accumulation of TOC1, thereby linking the activity of two proteins (Wang et al., 2010).

In the model, the transcription of *PRR9/PRR7* mRNA is transiently light-induced at dawn, as is the case for *PRR9* (Khanna et al., 2006). It is induced by CCA1/LHY and repressed by PRR5/TOC1 and the EC. The protein is degraded faster in the absence of light and inhibits the transcription of *CCA1/LHY*. The transcription of *PRR5/TOC1* is inhibited by CCA1/LHY and PRR5/TOC1. The protein is degraded differently in the light than in the dark, and inhibits the expression of all of the clock genes, including itself.

The degradation of PRR5 and TOC1 is known to be mediated by two other proteins, GI and ZTL. The degradation rate is not constant throughout the night. Instead, it starts out slow at dusk, when a large fraction of ZTL proteins are sequestrated by GI, and accelerates as the night progresses, when more ZTL is released from the complex (Fujiwara et al., 2008). In our model, we have greatly simplified that regulation, and only have different degradation rates in light and darkness. A more detailed description could potentially improve the description of the evening section of the clock; however, when we attempted to include such a description, we found that the simplest approximations actually worsened the fit of the model. We needed at least three additional equations and over a dozen parameters just to match the performance of the model version presented here. The resulting system had a slightly improved description of the phase of *PRR5/TOC1* but no major qualitative changes. Since adding this regulation increased the number of variables and parameters by over 30% each, we considered that we had reached the level of complexity where the cost of adding more details, in terms of ease of use, outweighed the benefits of a more accurate result.

It should be noted that several previous models described an activation cascade where PRR9 induces *PRR7* expression and PRR7 induces *PRR5* in turn, while *TOC1* was considered separately (Pokhilko et al., 2010, 2012, 2013). Such a cascade was initially hypothesized to exist because of the pattern of *PRR* expression during the day: the members of the family are sequentially expressed, with *PRR9* peaking shortly after dawn, followed by *PRR7* in the morning, *PRR5* in the evening, and *TOC1* at or after dusk (Makino et al., 2000; Matsushika et al., 2000). We have not included the PRR cascade in our model, for similar reasons to the ones outlined in Fogelmark and Troein (2014). First, the data on knockout and overexpressor lines does not support the hypothesis. The *prr9* knockout mutant has a very mild phenotype including almost wild-type levels of *PRR7* and *PRR5* (Farré et al., 2005), showing that *PRR9* can not be necessary for the expression of *PRR7/5*. Similarly, *PRR5* is not suppressed in the *prr7* mutant, making *PRR7* unnecessary for the expression of *PRR5* (Farré et al., 2005). Conversely, *PRR9-OX* (Matsushika et al., 2002) and *PRR7-OX* (Matsushika et al., 2007) lines do not have elevated levels of the next *PRR* in line, which would be expected if they were activators. Other studies have shown that while some of the PRRs bind each others promoters, they are strictly transcriptional repressors (Nakamichi et al., 2010).

#### 2.1.4. Acute light-induced activation: the protein P

The transcription of the morning genes *CCA1, LHY* and *PRR9* is induced upon transition from an extended period of darkness to light, through the action of dark-accumulating proteins: PHYTOCHROME INTERACTING FACTOR 3-LIKE 1 (PIL1) for *PRR9* (Khanna et al., 2006) and possibly PHYTOCHROME INTERACTING FACTOR 3 (PIF3) along with other unidentified agents for *CCA1* and *LHY* (Martínez-Garcia et al., 2000). In the model, those proteins are all represented by a non-clock regulated variable called P, which accumulates during dark periods and is rapidly degraded by light. The equation for P was taken directly from previously published models. During the building of our model, we examined whether replacing this variable with an explicitly clock-regulated one (similar to the PIF4/5 protein included in the hypocotyl growth module, see equations in the Supplementary Information) improved the description of the clock, and found virtually no difference.

### 2.2. Model equations

The model consists of nine ODEs (See Supplementary Materials). Eight equations describe the temporal evolution of the mRNA and protein levels of the main clock genes, grouped into four groups, each of which represents a pair of clock genes: CL (*CCA1* and *LHY*), P97 (*PRR9* and *PRR7*), P51 (*PRR5*, and *TOC1*), and EL (*ELF4*, and *LUX*). The ninth equation is the dark-accumulating protein P, introduced by Locke et al. (2005a) and used by subsequent models as a stand-in for PIL1, PIF3 and other proteins that induce the transcription of *CCA1, LHY*, and *PRR9* upon dark-to-light transition. All rates of protein synthesis, protein degradation, and mRNA degradation are linear. The rates of mRNA transcription are non-linear, and activations/inhibitions are modeled using Hill-type terms. The environmental light conditions are represented by the parameter L, which only takes the values 0 (lights off) or 1 (lights on). Light/dark cycles are thus modeled by square waves. The light is considered to be white light at a constant fluence rate of around 100 μmol.m^−2^.s^−1^. The model contains 34 parameters. Parameter values were obtained through automated optimization as described in the Section 5.

## 3. Results

The simple model captures the main features of the wild-type clock and the main clock mutants, in both entrained and free-running conditions. In contrast to earlier models of similar complexity, this model also responds well to unusual light cues, including extreme photoperiods and non-24 h light/dark cycles.

### 3.1. The wild-type clock

**Figure 2:**
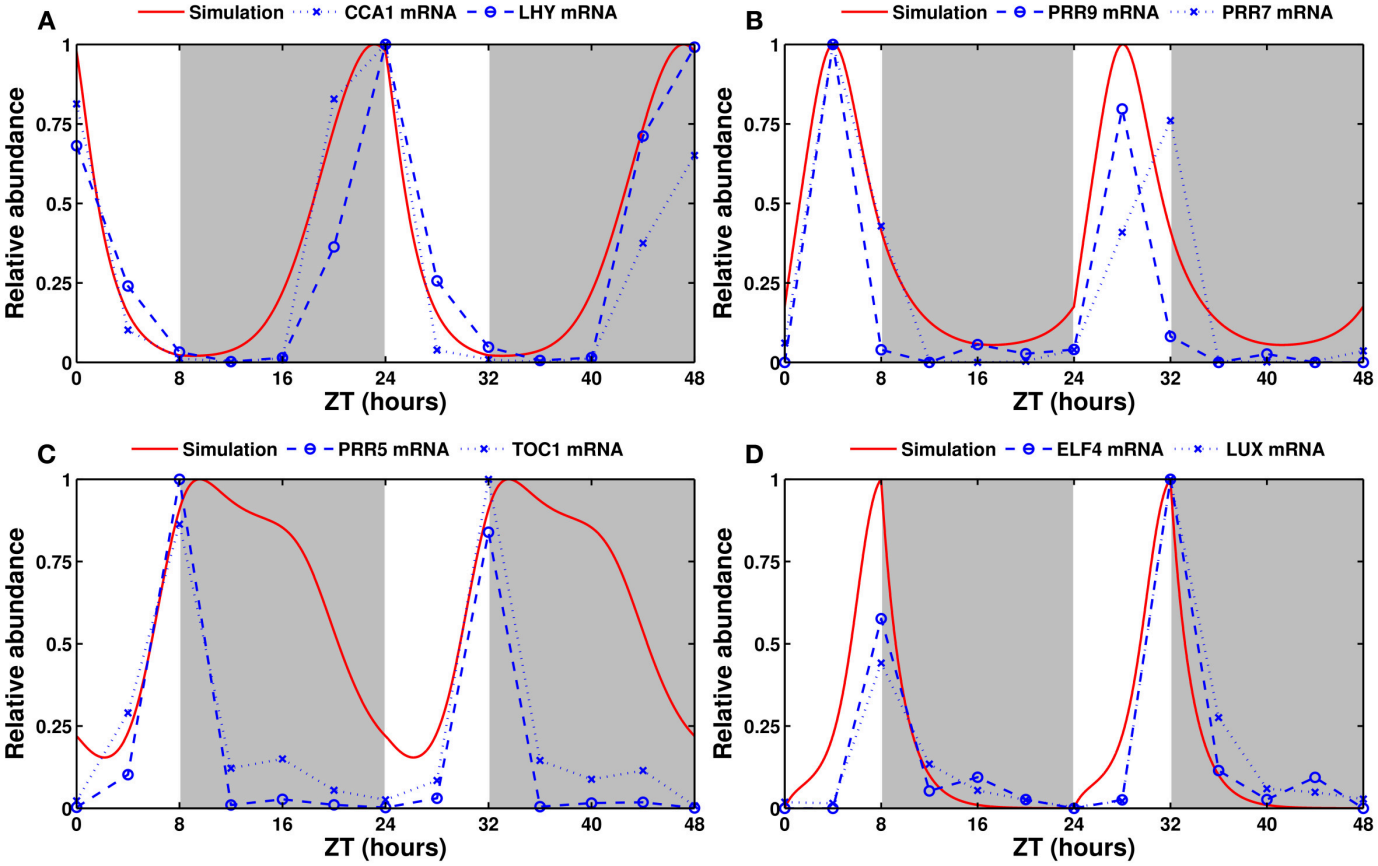
**Experimental and simulated expression profiles in short days (8L:16D)**. Experimentally measured (in blue) and simulated (in red) mRNA levels of **(A)** *CCA1* and *LHY*, **(B)** *PRR9* and *PRR7*, **(C)** *PRR5* and *TOC1*, and **(D)** *ELF4* and *LUX*. All values are normalized to their respective maximum. The experimental data was obtained by qRT-PCR (see Section 5).

We first present the time profiles, obtained after optimization, for the wild-type clock under various LD cycles. As shown in Figure 2, in short days (8L:16D), the simulated mRNA profiles of three of the four model components match the experimental data very closely. The expression level of *PRR5/TOC1* remains high much longer in simulations than in experiments. However, PRR5 and TOC1 proteins are known to persist well into the dark phase (Fujiwara et al., 2008; Kim et al., 2011), and the simulated PRR5/TOC1 protein (not shown) peaks much closer to its experimental counterparts (Kim et al., 2007). Furthermore, depending on the source, the reported phase of *TOC1* in the Columbia (Col-0) wild type in short days varies between Zeitgeber Time (ZT) 8 (our data, shown here) and ZT15 (Edwards et al., 2010). The source of this important variation is unknown, but may be related to growth conditions.

**Figure 3:**
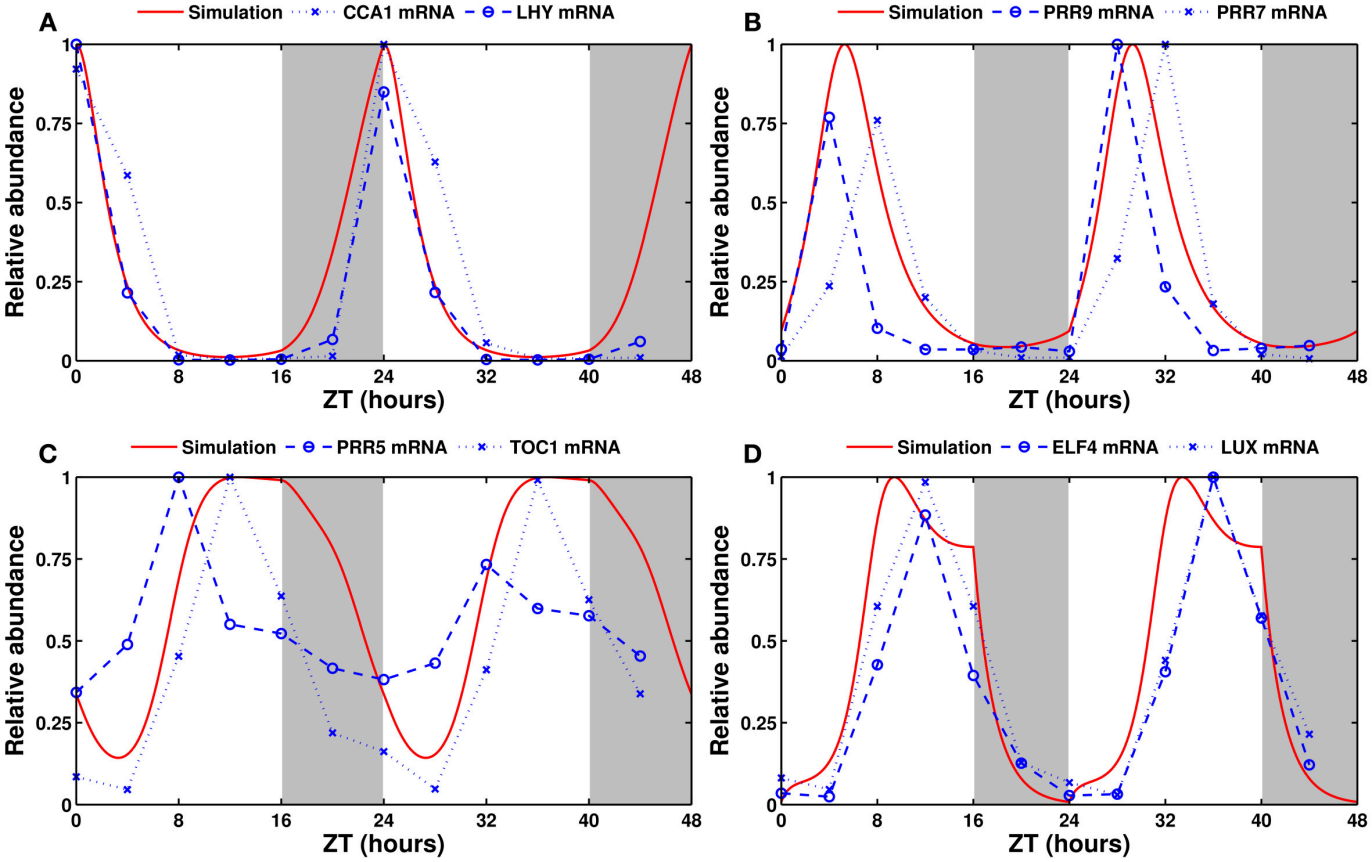
**Experimental and simulated expression profiles in long days (16L:8D)**. Experimentally measured (in blue) and simulated (in red) mRNA levels of **(A)** *CCA1* and *LHY*, **(B)** *PRR9* and *PRR7*, **(C)** *PRR5* and *TOC1*, and **(D)** *ELF4* and *LUX*. All values are normalized to their respective maximum. The experimental data was obtained from the DIURNAL database (Mockler et al., 2007).

The simulated expression of clock genes varies according to the entraining photoperiod, as observed experimentally. Figure 3 shows simulated and experimentally measured mRNA levels of the eight modeled clock genes in long days (16L:8D). Supplementary Figure 2 shows the same in the standard 12L:12D conditions. The model provides a good fit in all three cases.

**Figure 4:**
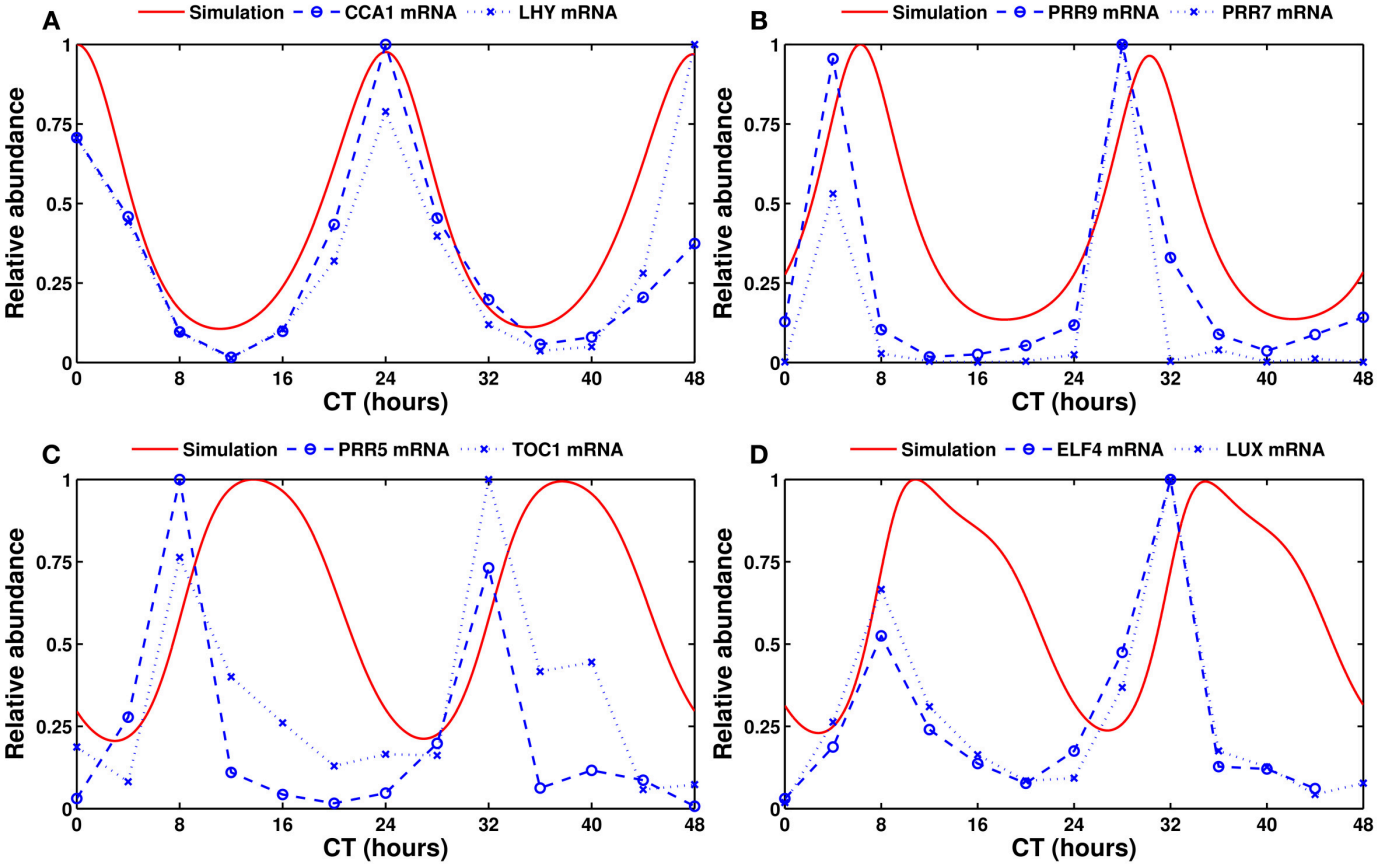
**Experimental and simulated expression profiles in continuous light conditions**. Experimentally measured (in blue) and simulated (in red) mRNA levels of **(A)** *CCA1* and *LHY*, **(B)** *PRR9* and *PRR7*, **(C)** *PRR5* and *TOC1*, and **(D)** *ELF4* and *LUX*. All values are normalized to their respective maximum. The experimental data was obtained by qRT-PCR (see Section 5).

Figure 4 shows that the model produces strong, sustained oscillations with a period of 24 h in continuous light. In continuous darkness, the period is 25.7 h (not shown). This reflects the fact that the free-running period is generally reported to be between 24 and 25 h in white light, and longer and more variable in darkness (see for example, Dalchau et al., 2011).

### 3.2. Clock mutants

In order to evaluate the ability of the model to reproduce the observed defects (defined as any change in gene expression levels, phase, or free-running period length) associated with a loss-of-function of the main clock genes, we looked at those features in single and double mutants. For three of the four gene pairs in the model, there are two single mutants with the same or very similar defects, and a double mutant with qualitatively similar but more pronounced version of the same phenotype.

The fourth pair, *ELF4* and *LUX*, is different: since they function together as part of the same protein complex instead of separately, their effects are not additive, and the loss of either one leads to the loss of the whole EC. Both single mutants are completely arrhythmic in continuous light. However, there exists a reduced function *ELF3* mutant (*elf3-12*), in which the clock is only mildly affected. This mutant has lower levels of nuclear ELF3 protein than the wild type, a light-dependent short period phenotype, and its growth- and clock-related defects are intermediate between the wild type and *elf3-1*, a null mutant. (Kolmos et al., 2011) Thus, we defined the *elf3-12* line as the weak EC mutant, similar to the single mutants for the other pairs, and the *elf3, lux*, and *elf4* null lines as the strong EC mutants, similar to the double mutants in other pairs.

**Figure 5:**
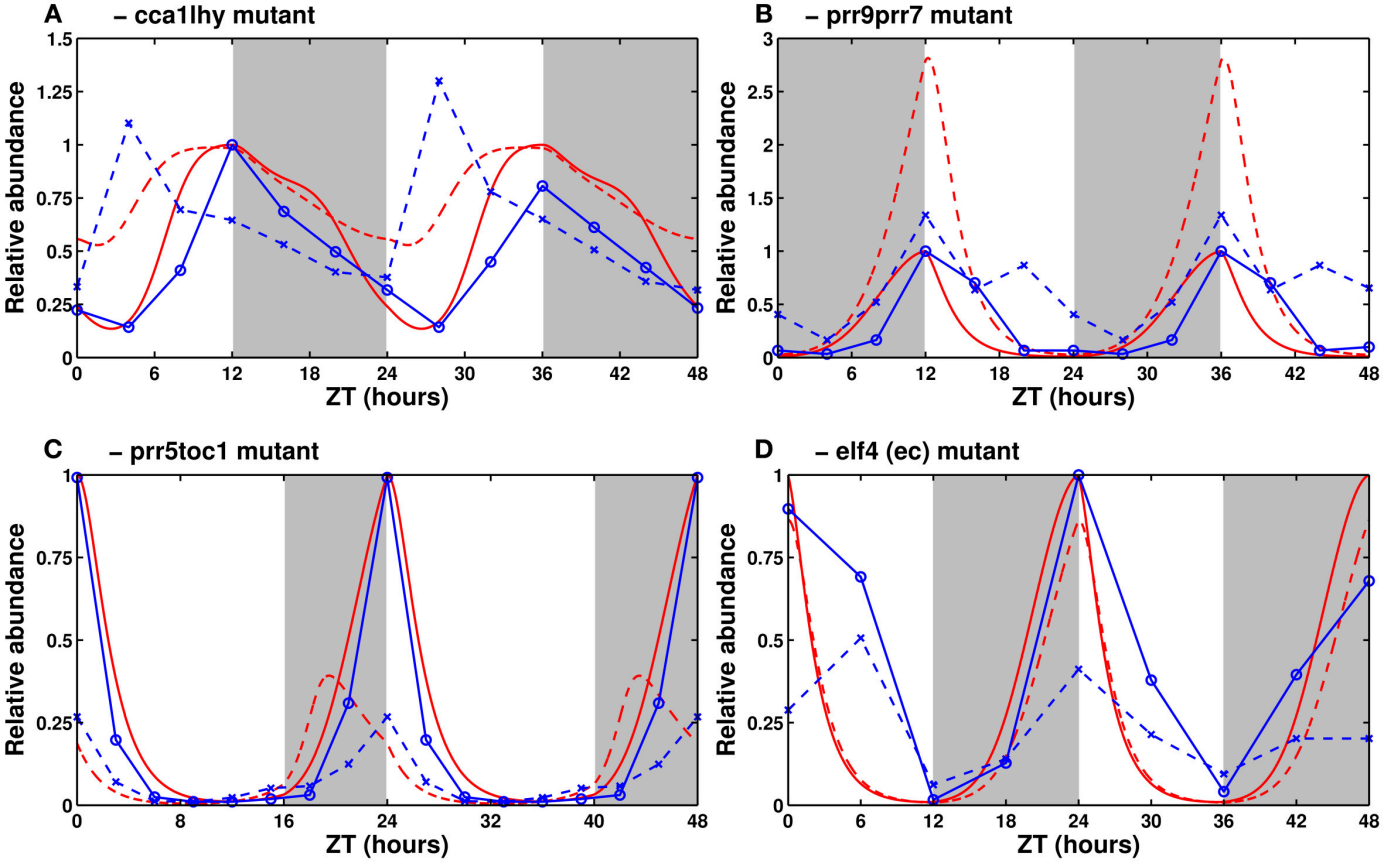
**Expression of clock genes in loss-of-function mutants**. Experimental (in blue) and simulated mRNA levels (in red) in various clock mutants (dashed lines) and their respective wild types (solid lines). **(A)** Experimental *TOC1* and simulated *PRR5/TOC1* expression in the *cca1lhy* mutant. Data from Mizoguchi et al. (2002). **(B)** *CCA1/LHY* expression in the *prr9prr7* mutant. Data from Farré et al. (2005). **(C)** Experimental *LHY* and simulated *CCA1/LHY* expression in the *prr5toc1* double mutant. Data from Ito et al. (2008). **(D)** Experimental *CCA1* and simulated *CCA1/LHY* expression in the *elf4* mutant. Data from McWatters et al. (2007). All values are normalized to the maximum of the respective wild type. The sources for **(B,C)** have, respectively, 24 and 21 h worth of data, duplicated here for the sake of homogeneity between panels.

Simulated single mutants were produced by dividing the relevant mRNA synthesis rate by 2. For simulated double mutants, the synthesis rate was divided by 10. The results of the double mutant simulations are shown in Figure 5. Mutant simulations in continuous light conditions can be found in Supplementary Figure 3.

#### 3.2.1. *cca1, lhy*, and *cca1lhy* mutants

Experimentally, in light/dark cycles, *CCA1* and *LHY* set the phase of most evening genes by preventing their expression in the hours directly after dawn. In *cca1, lhy*, and *cca1lhy* mutants, this effect is diminished or lost, leading the evening-phased genes (including *ELF4, LUX, TOC1*, and *PRR5*) to rise early. In continuous light, the single mutants have a short period while the double mutant is generally reported to be arrhythmic (Alabadí et al., 2002; Mizoguchi et al., 2002).

Figure 5A shows simulated levels of *PRR5/TOC1* in the double mutant in light/dark conditions. Its elevated expression level, especially in the morning, is correctly reproduced. In continuous light (Supplementary Figure 3A), the simulated single mutant has sustained short-period oscillations, while the simulated double mutant is arrhythmic, which matches the experimentally reported behavior.

#### 3.2.2. *prr9, prr7*, and *prr9prr7* mutants

Experimentally, the *prr9* and *prr7* single mutants have only very slight defects, both in period length (slightly long, 25 to 26 h Farré et al., 2005; Salomé and McClung, 2005) and in clock gene expression levels. The *prr9prr7* double mutant, however, has a strong (temperature-dependent) phenotype, with measured period lengths of around 32 h at 20/22°C and exceeding 35 h at higher temperatures (Salomé et al., 2010). In light/dark cycles, it has elevated levels of *CCA1* and *LHY*, which fail to return to baseline in the morning, thus clamping the expression of evening genes very low for most of the day (Farré et al., 2005).

Figure 5B shows *CCA1/LHY* expression in the simulated double mutant. The mRNA levels are elevated, as observed experimentally, though the magnitude of the effect is larger in the simulation. In continuous light, lowering the rate of *PRR9/PRR7* synthesis correctly lengthens the period, but the system becomes completely arrhythmic if the synthesis rate is set to zero. This behavior is reminiscent of the *prr9prr7prr5* triple mutant (Nakamichi et al., 2005). A possible explanation for the exaggerated features of the simulated double mutant is the partial redundancy between PRR7 and PRR5, which the model does not account for, and which is probably sufficient to sustain clock function in the *prr9prr7* double mutant.

#### 3.2.3. *prr5, toc1*, and *prr5toc1* mutants

Experimentally, the *prr5* and *toc1* mutants have short periods, and the *prr5toc1* double mutant is shorter than each single mutant, although the magnitude of the synergetic effect is lower than for *CCA1/LHY* and *PRR9/PRR7* (Ito et al., 2008). All three mutants have low *CCA1* and *LHY* expression. This is seemingly in contradiction with the role of the PRRs as inhibitors of *CCA1/LHY* expression and is the reason why *TOC1* was initially thought to be an activator (Locke et al., 2005a).

As seen in Figure 5C, the model correctly reproduces the low levels of *CCA1/LHY*. That effect is due to the indirect activation of *CCA1/LHY via* the repression of *PRR9/PRR7* overpowering the direct inhibition. The phase of *CCA1/LHY* peak expression is early in the model, due to the loss of the night-phased PRR5 and TOC1 proteins allowing the morning genes to rise early. A possible explanation for the difference between the simulation and experiments lies in the lack of a detailed description of the regulation of PRR5/TOC1 protein stability by GI/ZTL. The model building section discusses our reasons for not adding this regulation.

#### 3.2.4. *elf4* and *lux* mutants

Experimentally, the effects of the *elf4* and *lux* mutations are the same rather than being additive. Both mutants are arrhythmic in constant light, with the clock arresting around dusk, when the level of all of the *PRRs* are elevated, and *CCA1* and *LHY* are almost completely suppressed (Onai and Ishiura, 2005; McWatters et al., 2007).

In simulations, lowering the synthesis rate of the EC shortens the free-running period. This is coherent with the reported short period of reduced-function *elf3* lines, which presumably have reduced EC levels. The null mutant, with a synthesis rate set to zero, is arrhythmic in free-running conditions. With the parameters presented here, the simulated evening complex mutant has only slightly lower levels of *CCA1/LHY* than the wild-type in light/dark cycles (Figure 5D). However, *CCA1/LHY* expression correctly drops to very low levels in continuous light (Supplementary Figure 2D). The free-running period is short in weak mutants, and the oscillations rapidly damp to undetectable levels in strong mutants.

### 3.3. Entrainment by light/dark cycles

In our model, light affects every gene, whether at the transcriptional, translational, or post-translational levels. Those multiple light-sensing points are all backed up by experimental data, as described in our model building section, and allow for complex responses to a wide range of light conditions.

#### 3.3.1. Dawn and dusk sensitivity

Both at the experimental and simulation levels, changing the length of the photoperiod leads to changes in the phase and amplitude of expression of many clock genes. The relationship between the phase of maximum (or minimum) expression of a gene and the photoperiod can be characterized and even quantified using the concept of dawn- and dusk-sensitivity (Edwards et al., 2010; Dixon et al., 2014). A gene is said to be dawn-sensitive if its phase is completely independent of the photoperiod (i.e., always at the same time relative to dawn). Conversely, the phase of a dusk-sensitive gene is entirely dependent on the photoperiod (i.e., always at the same time relative to dusk). The clock genes *CCA1, LHY, PRR9*, and *TOC1* have been systematically characterized in photoperiods ranging from 3 to 18 h and have more complex responses than pure dawn- or dusk-tracking (Edwards et al., 2010). Data for other clock genes is generally only available in 8-, 12-, and 16-h photoperiods.

**Figure 6:**
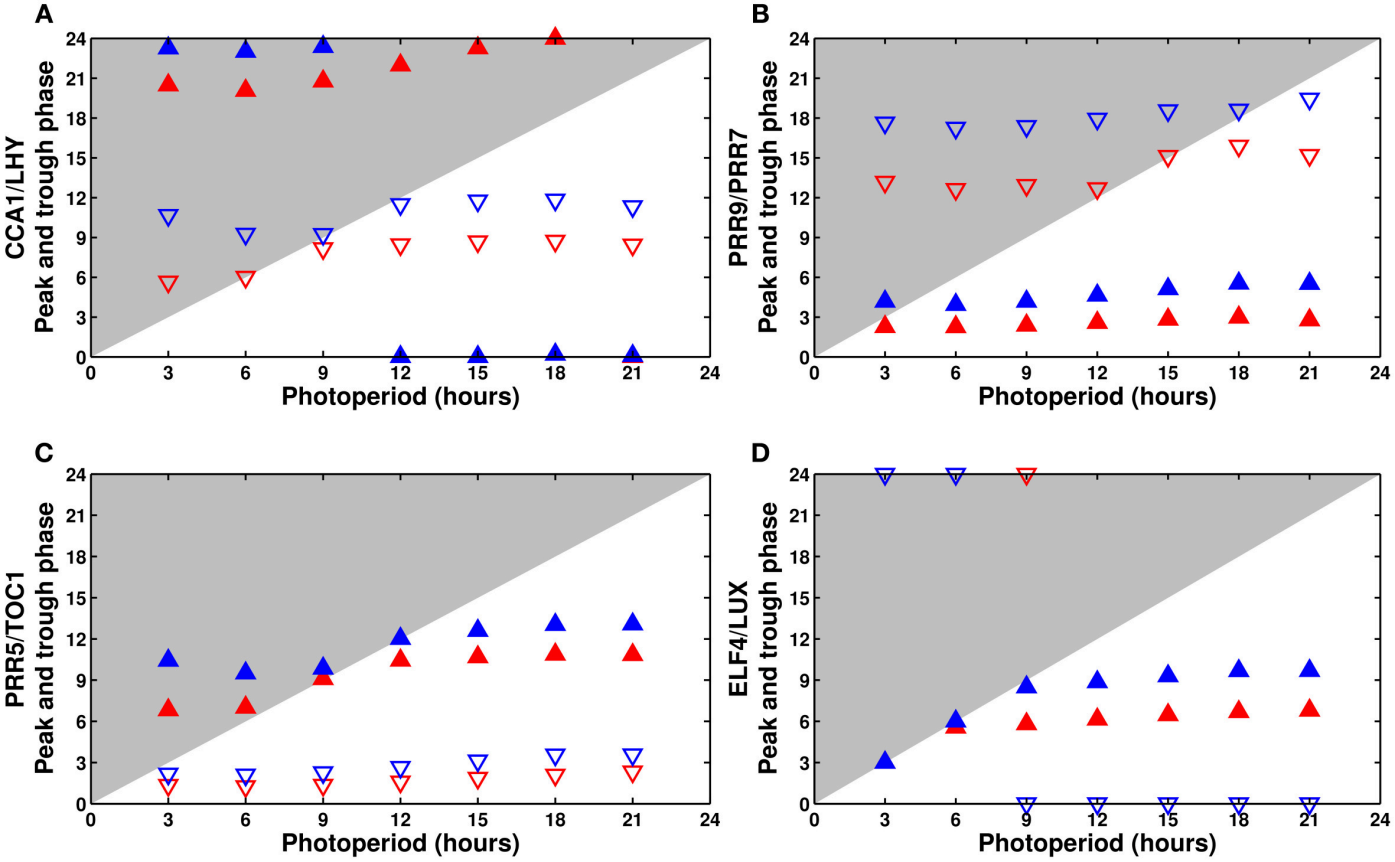
**Phase dependence on the photoperiod: (A) CCA1/LHY, (B) PRR9/PRR7, (C) PRR5/TOC1, and (D) ELF4/LUX**. Phase of the peak (filled upward triangles) and trough (empty downward triangles) of the model components as a function of the photoperiod. The wild type is in blue, the *cca1* mutant in red. The typical phase advance of the mutant is clearly apparent.

The response of the model variables in photoperiods ranging from 3 to 21 h in increments of 3 h is shown in Figure 6. Although the exact phase tends to be slightly off, the qualitative behavior of the model largely matches the findings of Edwards et al. (2010).

Experimentally, *CCA1* and *LHY* are almost completely dawn-sensitive, peaking shortly after lights on in all photoperiods. The model correctly predicts almost perfect dawn sensitivity for *CCA1/LHY* (Figure 6A), though the variable slightly overanticipates the end of the night in very short days.

Edwards et al. (2010) reported that *PRR9* is partially dusk-sensitive, but they did not characterize its homolog *PRR7*. In the model, *PRR9/PRR7* has a small but noticeable degree of dusk sensitivity, which agrees with the reported behavior of *PRR9* (Figure 6B).

The simulated *PRR5/TOC1* (Figure 6C) has the most complex response, with a phase that first gets earlier, then later as the photoperiod becomes longer. The same behavior is observed experimentally with *TOC1*. Unfortunately, no full data is available for *PRR5*.

Finally, although neither *ELF4* nor *LUX* were systematically measured in all photoperiods, their simulated response (Figure 6D) is coherent with the limited data available from the literature, which generally gives their phase of peak expression as ZT8 in 8L:16D conditions, ZT11-12 in 12L:12D, and around ZT12-13 in 16L:8D. In our simulations, *ELF4/LUX* displays a light-dependent behavior, tracking dusk perfectly in short days then becoming mostly dawn-sensitive in longer days.

In addition to the wild type, Figure 6 shows the same analysis for the simulated *cca1* or *lhy* single mutant. Plotting the phase of all of the clock components can highlight the defects of each mutant, in this case a general phase advance of the whole clock caused by the premature release of the evening genes.

#### 3.3.2. Entrainment and release

**Figure 7:**
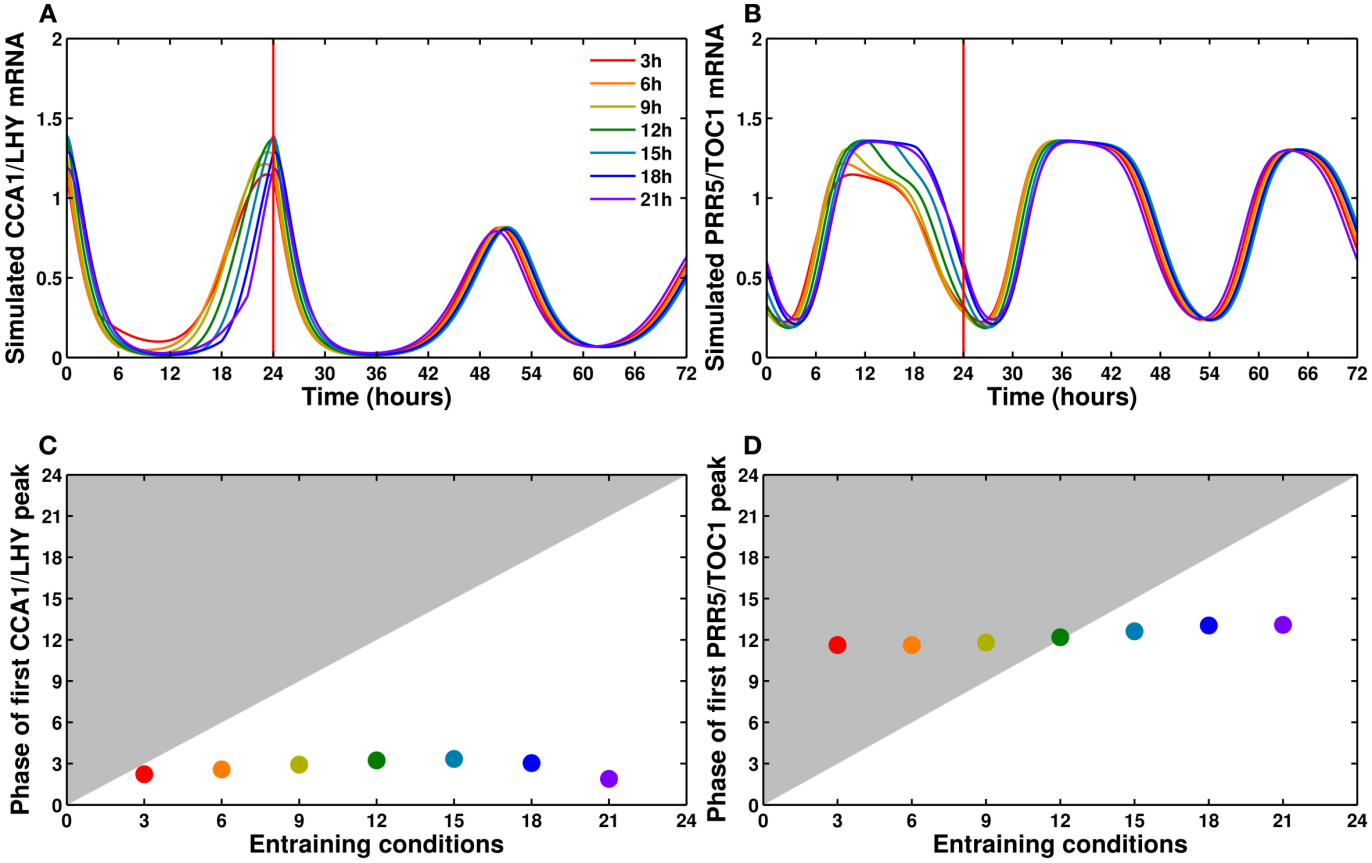
***CCA1/LHY* and *PRR5/TOC1* expression profiles in different photoperiods**. **(A,B)** Simulated profiles of *CCA1/LHY* **(A)** and *PRR5/TOC1* **(B)**: one cycle in photoperiods ranging from 3 to 21 h in increments of 3 h, followed by release into continuous light at time 24. The colors go from red for a 3 h photoperiod to purple for a 21 h photoperiod. The time of the first peak in free-running conditions is almost independent of the entraining photoperiod. **(C,D)** Relationship between the entraining photoperiod and the phase of the first free-running peak.

As discussed above, our model can be reliably entrained to a 24 h cycle regardless of the length of the light period. Figure 7 shows the simulated time courses for *CCA1/LHY* (Figure 7A) and *PRR5/TOC1* (Figure 7B) expression during one light/dark cycle (following several cycles of adaptation, not shown) in photoperiods ranging from 3 to 21 h, followed by release into continuous light. As we can see on those panels, and plotted on Figures 7C,D, the phase of the first peak of expression after release into continuous light is largely independent of the preceding entraining conditions. This correlates well with the experimental data (Edwards et al., 2010) and in contrast to several previous models. While the more recent models (Pokhilko et al., 2012, 2013; Fogelmark and Troein, 2014) have the same feature, the earlier ones either lack the capacity to adapt to changing photoperiods (Locke et al., 2005a,b) or show an important correlation between the phase of the free-running clock and the length of the last photoperiod before release (Locke et al., 2006; Edwards et al., 2010).

This feature of our model is coherent with the existing literature, as it includes a complex network structure and multiple light inputs, both of which have been shown to be important factors in the ability of a system to respond to light cues (Troein et al., 2011; Dixon et al., 2014; Ohara et al., 2015).

#### 3.3.3. Exotic light cues: non-24 h cycles and skeleton photoperiods

An oscillator has a range of entrainment, that is, a range of periods it can be entrained to. Frequency demultiplication is a particular phenomenon that happens when the clock is subjected to a short cycle that is outside of its range of entrainment, but has a frequency that is close to an integer multiple of the free-running frequency. In such a case, the clock can skip cycles and entrain not to the short period but to a multiple of it. The wild-type Arabidopsis clock has been shown to oscillate with a 24 h period for at least several cycles following a switch from standard 24 h (12L:12D) conditions to a 12 h (6L:6D) (Kolmos et al., 2011; Anwer et al., 2014) or 8 h (4L:4D) cycle. (Nozue et al., 2007) In contrast, clock mutants with severe defects, such as the arrhythmic *CCA1-OX* and *elf3*, synchronize to the short cycle (Nozue et al., 2007).

**Figure 8:**
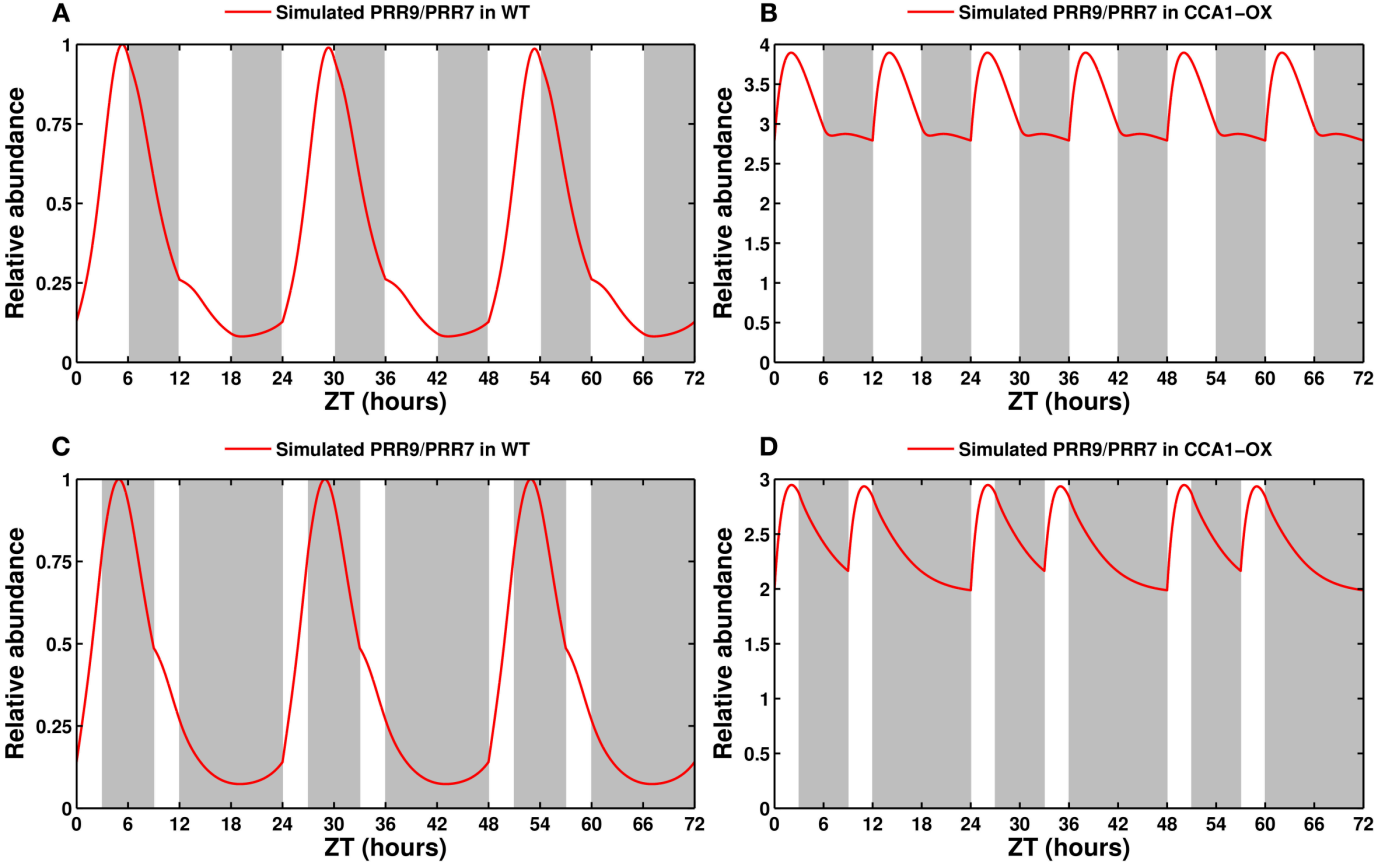
**Entrainment by non-24 h cycles and skeleton photoperiods**. **(A)** Entrainment of the wild type clock by a 12 h cycle (6L:6D). The clock oscillates with a 24 h, not 12 h, period. **(B)** Response of a CCA1-overexpressing line in a 12 h cycle. This arrhythmic line entrains to the short cycle. **(C)** Entrainment of the wild type clock by a skeleton photoperiod (3L:6D:3L:12D). The functional clock entrains to the 24 h cycle. **(D)** Response of the CCA1-OX line to a skeleton photoperiod.

The model is fully capable of reproducing this behavior: as shown in Figures 8A,B, in 6L:6D cycles, the wild type clock entrains to a 24 h period. Heavily affected/arrhythmic mutants, such as the CCA1-OX line shown here, or the *cca1lhy* double mutant (not shown), immediately entrain to the short cycle. Mutants with mild to moderate defects (not shown) initially retain a 24 h period before transitioning to the short cycle.

Another hallmark of a functional circadian clock is the ability to be entrained by skeleton photoperiods, in which the organism is maintained in darkness except for pulses of light at dawn and dusk. Experimentally, the clock in *A. thaliana* will oscillate with a 24 h period provided that the dawn and dusk light pulses are at least 3 h long (Pokhilko et al., 2010). Figure 8C shows that the simulated wild type clock readily locks onto a 24 h cycle, as reported, while the arrhythmic CCA1-OX line, in Figure 8D, is predicted to simply respond to the light cues.

### 3.4. Clock output: hypocotyl length

One of the motivations for developing clock models, aside from understanding the core circadian oscillator itself, is to investigate the many clock-controlled metabolic and physiological aspects. To ensure the ability of our minimal model to capture the general features of such processes, we have developed a small output module to model hypocotyl growth.

Hypocotyl growth is gated by light and the circadian clock through a coincidence mechanism. The clock controls the expression of the partially redundant genes *PIF4* and *PIF5*, two major activators of hypocotyl elongation. Light degrades the PIF4/5 proteins through a phytochrome-mediated mechanism. In long days, the EC is active throughout the night, and *PIF4/5* are only expressed during the light period. Under these conditions, the proteins cannot accumulate, and growth is suppressed. In shorter days, the EC is fully degraded before the end of the night, allowing *PIF4/5* transcription and protein accumulation before dawn, and thus enabling hypocotyl growth.

This output module keeps the same minimalist approach as the rest of the clock, consisting of three equations and nine parameters. The three modeled variables are *PIF4/5* mRNA (combined as a single variable, as with the clock genes), which is inhibited by the EC, the PIF4/5 protein, which is rapidly degraded by light, and hypocotyl length, which starts at zero and accumulates with time.

**Figure 9:**
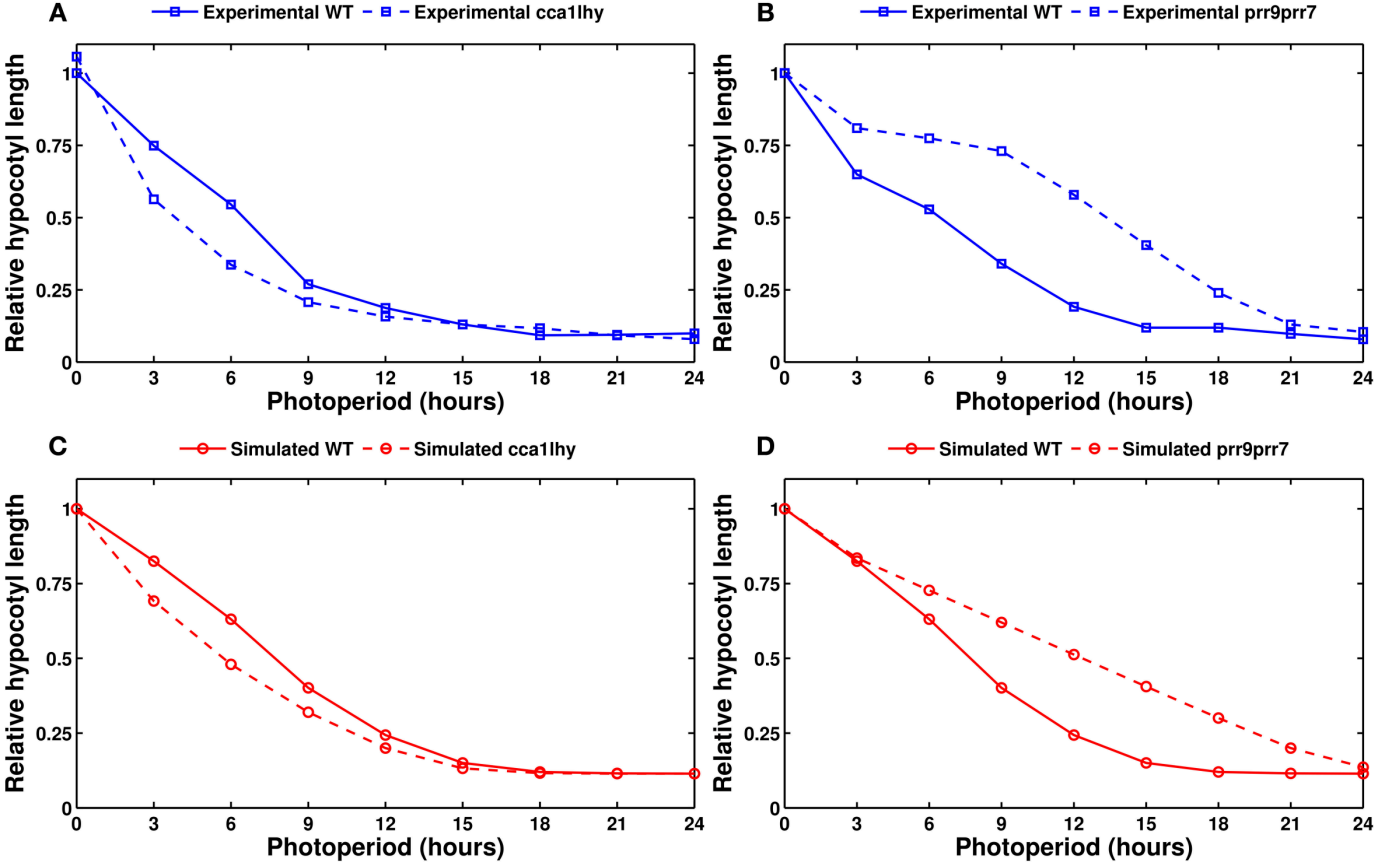
**Modeling hypocotyl growth**. All panels show the measured or simulated hypocotyl length after 5 days of growth, relative to the length of the wild type in complete darkness. **(A)** Experimental data for the wild type (solid line) and the *cca1lhy* double mutant (dashed line). **(B)** Experimental data for the wild type (solid line) and the *prr9prr7* double mutant (dashed line). **(C)** Simulated data for the wild type (solid line) and *cca1lhy* (dashed line). **(D)** Simulated data for the wild type (solid line) and *prr9prr7* (dashed line). Experimental data from Niwa et al. (2009).

Figure 9 shows the experimentally measured and simulated length after 5 days of growth as a function of the photoperiod, in the wild type and two mutants. The model correctly captures the two domains of activity in the wild type, with hypocotyls remaining short in long photoperiods before growing as a mostly linear function of day length. The model also correctly predicts the growth defects of the *cca1lhy* and *prr9prr7* mutants. The simulated *elf4/lux* mutant (not shown) has a long hypocotyl, similar to *prr9prr7*, which fits the experimental evidence. However, the model incorrectly predicts a short hypocotyl phenotype for *prr5toc1* (also not shown), which is reported to have an elongated hypocotyl (Ito et al., 2008).

## 4. Discussion

Our minimal model is able to reproduce many of the key features of the *A. thaliana* circadian clock, including clock mutant phenotypes and light responses. It demonstrates that the molecular mechanism of the plant oscillator can be described by a reduced set of equations. It highlights the importance of multiple light sensing mechanisms and offers a more qualitative but more tractable alternative to recent detailed models.

The number of equations and parameters in our model (9 equations and 34 parameters) is similar to that of the very first one-loop model proposed by Locke et al. (2005a) (7 equations and 29 parameters). Nonetheless, the significantly different network structure allows us to capture a much wider range of behaviors. Locke et al. (2005a) included only two genes, one feedback loop, and one light entry point, and could only reproduce the behavior of the wild-type clock. In contrast, our model has four groups of genes, multiple interlocked loops and multiple light inputs, allowing it to simulate various clock mutants and respond to a wide range of light cues. To further study these responses, the role of multiple light inputs in the seasonal adaptation could be explored via a systematic analysis of the entrainment in different photoperiods and T-cycles (Schmal et al., 2015). The robustness of the circadian oscillations in presence of both molecular and environmental variability can readily be investigated through stochastic simulations (Guerriero et al., 2012, 2014).

The ability of our minimal model to respond to various photoperiods is similar to that of the most recent clock models, published by Pokhilko et al. (2013) (32 equations and 133 parameters) and Fogelmark and Troein (2014) (35 equations and 119 parameters), which have three to four times as many components. Those detailed models are useful to dissect the core regulatory mechanism of the clock but are less convenient to manipulate. Besides the decrease of the computational cost for the optimization, methods such as sensitivity and bifurcation analyses can be applied more easily on simpler models (See Supplementary Figure 5).

While the current results are more qualitative than quantitative, the model could be expanded to either split the gene pairs into individual variables or include more detailed descriptions of various effects, especially of post-translational regulations affecting protein stability and activity. This would allow a more quantitative description of the clock response to environmental stimuli, at the cost of reintroducing complexity into the system.

Nonetheless, our simple model is capable of driving an equally simple output module to reproduce the experimentally measured hypocotyl length of the wild type plant and predict that of several mutant lines. This demonstrates the potential use of our model in studying both the core circadian clock and clock-dependent processes. The model could also be coupled to more output modules, including clock-controlled slave oscillators (as done by Schmal et al., 2013). More generally, processes like hypocotyl and root growth, flowering, photosynthesis, nutrient uptake, and sugar metabolism are known to be tightly regulated by the circadian clock. As shown here for hypocotyl growth and in Seaton et al. (2015) for both hypocotyl growth and flowering time, output pathways can be coupled to the core clock to predict the response to various photoperiods. Modeling these processes requires a clock model that is capable of reproducing key features of the circadian clock without an explicit description of all genes and regulations. Our model constitutes a suitable tool for this purpose by simulating entrainment under various light–dark conditions and the main clock mutants.

## 5. Materials and methods

### 5.1. Model simulation and optimization

All simulations were done in MATLAB and XPPAUT (Ermentrout, 2002). Bifurcation diagrams were made in XPPAUT (Schmal et al., 2014). The model was developed with the MATLAB toolbox SBToolBox2 (Schmidt and Jirstrand, 2006). All integrations were performed using the CVODE solver bundled in the toolbox. Parameter estimation was done using the genetic algorithm and simulated annealing algorithm from the MATLAB Global Optimization Toolbox.

During the optimization process, the clock was simulated in 8L:16D conditions for a total of 384 h, then released into free-running conditions for 300 h. The purpose of the first 360 h of simulation is to allow the system to reach its limit cycle; the scoring algorithm used only the last light/dark cycle to compute the score in entrained conditions. Similarly, the first 100 h in free-running conditions were discarded, as they contain transient effects, and the score was computed using the last 200 h of simulations. The wild type and the four single mutants (obtained by halving the relevant mRNA synthesis rates) were simulated in 8L:16D and continuous light. Additionally, the wild type was simulated in continuous darkness.

Because the model is very simple and meant to be more qualitative than quantitative, the optimization function was similar to the one defined in Locke et al. (2005a), using arbitrary constraints on period length and amplitude rather than experimental data sets. The cost function is described in more detail in the Supplementary Information.

### 5.2. Plant growth conditions

*A. thaliana* Columbia-0 (Col-0) were grown in hydroponic system as described in Hermans et al. (2010a,b). Plants were grown in a chamber with 8 h light (100 μmol photon m^−2^ s^−1^)/16 h dark photoperiod at 21°C. After 5 weeks, plants were maintained in the same (8L:16D) or transferred to continuous (L:L) light conditions. Root and shoot organs were harvested every 4 h and immediately flash frozen in liquid nitrogen. Organs from three individual plants were pooled and ground prior to RNA extraction.

### 5.3. RNA extraction and qPCR assay

Total RNA was extracted and purified using Aurum total RNA mini kit (Bio-Rad). After NanoDrop RNA quantification (Thermo Scientific), reverse transcription was done with the GoScript Reverse cDNA Synthesis kit (Promega). Quantitative PCR (qPCR) reactions were performed in a final volume of 10 μl using VeriQuest SYBR Green qPCR Master Mix (Affymetrix) with the PikoReal Real-Time PCR System (Thermo Scientific): preincubation at 95°C for 7 min, 40 cycles at 95°C for 15 s, 60°C for 60 s followed by melting curves. Primers are listed in Supplementary Table 3. Levels of *CYCLIN-DEPENDENT KINASE A-1 (CDKA-1)* and *UBIQUITIN 10 (UBQ10)* were used as normalization factors. Data present the transcript levels of three pooled organs, each sample being assessed by three technical replicates.

## Author contributions

JD, JL, and DG conceived the model. JD performed the numerical simulations. CH and QX grew and harvested the plants. QX performed the qPCR. All authors analyzed the results. JD wrote the manuscript. All authors read and approved the manuscript.

### Conflict of interest statement

The authors declare that the research was conducted in the absence of any commercial or financial relationships that could be construed as a potential conflict of interest.

## References

[B1] Akman O. E. Watterson S. Parton A. Binns N. Millar A. J. Ghazal P. (2012). Digital clocks: simple Boolean models can quantitatively describe circadian systems. J. R. Soc. Interf. 9, 2365–2382. 10.1098/rsif.2012.0080 22499125 PMC3405750

[B2] Alabadí D. Oyama T. Yanovsky M. J. Harmon F. G. Más P. Kay S. A. (2001). Reciprocal regulation between TOC1 and LHY/CCA1 within the Arabidopsis circadian clock. Science 293, 880–883. 10.1126/science.1061320 11486091

[B3] Alabadí D. Yanovsky M. J. Más P. Harmer S. L. Kay S. A. (2002). Critical role for CCA1 and LHY in maintaining circadian rhythmicity in Arabidopsis. Curr. Biol. 12, 757–761. 10.1016/S0960-9822(02)00815-1 12007421

[B4] Anwer M. U. Boikoglou E. Herrero E. Hallstein M. Davis A. M. Velikkakam James G.. (2014). Natural variation reveals that intracellular distribution of ELF3 protein is associated with function in the circadian clock. eLife 3:e02206. 10.7554/eLife.02206 24867215 PMC4071560

[B5] Barak S. Tobin E. M. Andronis C. Sugano S. Green R. M. (2000). All in good time: the Arabidopsis circadian clock. Trends Plant Sci. 5, 517–522. 10.1016/S1360-1385(00)01785-4 11120473

[B6] Bolouri Moghaddam M. R. Van den Ende W. (2013). Sweet immunity in the plant circadian regulatory network. J. Exp. Bot. 64, 1439–1449. 10.1093/jxb/ert046 23564957

[B7] Corellou F. Schwartz C. Motta J.-P. Djouani-Tahri E. B. Sanchez F. Bouget F.-Y. (2009). Clocks in the green lineage: comparative functional analysis of the circadian architecture of the picoeukaryote ostreococcus. Plant Cell 21, 3436–3449. 10.1105/tpc.109.068825 19948792 PMC2798331

[B8] Dalchau N. (2012). Understanding biological timing using mechanistic and black-box models. New Phytol. 193, 852–858. 10.1111/j.1469-8137.2011.04004.x 22212235

[B9] Dalchau N. Baek S.-J. Briggs H. M. Robertson F. C. Dodd A. N. Gardner M. J.. (2011). The circadian oscillator gene GIGANTEA mediates a long-term response of the *Arabidopsis thaliana* circadian clock to sucrose. Proc. Natl. Acad. Sci. U.S.A. 108, 5104–5109. 10.1073/pnas.1015452108 21383174 PMC3064355

[B10] Dixon L. E. Hodge S. K. van Ooijen G. Troein C. Akman O. E. Millar A. J. (2014). Light and circadian regulation of clock components aids flexible responses to environmental signals. New Phytol. 203, 568–577. 10.1111/nph.12853 24842166 PMC4286021

[B11] Dixon L. E. Knox K. Kozma-Bognar L. Southern M. M. Pokhilko A. Millar A. J. (2011). Temporal repression of core circadian genes is mediated through EARLY FLOWERING 3 in *Arabidopsis*. Curr. Biol. 21, 120–125. 10.1016/j.cub.2010.12.013 21236675 PMC3028277

[B12] Dodd A. N. Belbin F. E. Frank A. Webb A. A. R. (2015). Interactions between circadian clocks and photosynthesis for the temporal and spatial coordination of metabolism. Front. Plant Sci. 6:245. 10.3389/fpls.2015.00245 25914715 PMC4391236

[B13] Dodd A. N. Salathia N. Hall A. Kévei E. Tóth R. Nagy F.. (2005). Plant circadian clocks increase photosynthesis, growth, survival, and competitive advantage. Science 309, 630–633. 10.1126/science.1115581 16040710

[B14] Doyle M. R. Davis S. J. Bastow R. M. McWatters H. G. Kozma-Bognár L. Nagy F.. (2002). The *ELF4* gene controls circadian rhythms and flowering time in *Arabidopsis thaliana*. Nature 419, 74–77. 10.1038/nature00954 12214234

[B15] Edwards K. D. Akman O. E. Knox K. Lumsden P. J. Thomson A. W. Brown P. E.. (2010). Quantitative analysis of regulatory flexibility under changing environmental conditions. Mol. Syst. Biol. 6:424. 10.1038/msb.2010.81 21045818 PMC3010117

[B16] Ermentrout B. (2002). Simulating, Analyzing, and Animating Dynamical Systems: A Guide to XPPAUT for Researchers and Students. Philadelphia: SIAM

[B17] Farré E. M. Harmer S. L. Harmon F. G. Yanovsky M. J. Kay S. A. (2005). Overlapping and distinct roles of PRR7 and PRR9 in the Arabidopsis circadian clock. Curr. Biol. 15, 47–54. 10.1016/j.cub.2004.12.067 15649364

[B18] Farré E. M. Kay S. A. (2007). PRR7 protein levels are regulated by light and the circadian clock in Arabidopsis. Plant J. 52, 548–560. 10.1111/j.1365-313X.2007.03258.x 17877705

[B19] Fogelmark K. Troein C. (2014). Rethinking transcriptional activation in the Arabidopsis circadian clock. PLoS Comput. Biol. 10:e1003705. 10.1371/journal.pcbi.1003705 25033214 PMC4102396

[B20] Fujiwara S. Wang L. Han L. Suh S.-S. Salomé P. A. McClung C. R.. (2008). Post-translational regulation of the Arabidopsis circadian clock through selective proteolysis and phosphorylation of pseudo-response regulator proteins. J. Biol. Chem. 283, 23073–23083. 10.1074/jbc.M803471200 18562312

[B21] Gendron J. M. Pruneda-Paz J. L. Doherty C. J. Gross A. M. Kang S. E. Kay S. A. (2012). Arabidopsis circadian clock protein, TOC1, is a DNA-binding transcription factor. Proc. Natl. Acad. Sci. U.S.A. 109, 3167–3172. 10.1073/pnas.1200355109 22315425 PMC3286946

[B22] Green R. M. Tingay S. Wang Z.-Y. Tobin E. M. (2002). Circadian rhythms confer a higher level of fitness to Arabidopsis plants. Plant Physiol. 129, 576–584. 10.1104/pp.004374 12068102 PMC161679

[B23] Guerriero M. L. Akman O. E. van Ooijen G. (2014). Stochastic models of cellular circadian rhythms in plants help to understand the impact of noise on robustness and clock structure. Front. Plant Sci. 5:564. 10.3389/fpls.2014.00564 25374576 PMC4204444

[B24] Guerriero M. L. Pokhilko A. Fernández A. P. Halliday K. J. Millar A. J. Hillston J. (2012). Stochastic properties of the plant circadian clock. J. R. Soc. Interf. 9, 744–756. 10.1098/rsif.2011.0378 21880617 PMC3284129

[B25] Haydon M. J. Mielczarek O. Robertson F. C. Hubbard K. E. Webb A. A. R. (2013). Photosynthetic entrainment of the *Arabidopsis thaliana* circadian clock. Nature 502, 689–692. 10.1038/nature12603 24153186 PMC3827739

[B26] Haydon M. J. Román Á. Arshad W. (2015). Nutrient homeostasis within the plant circadian network. Front. Plant Sci. 6:299. 10.3389/fpls.2015.00299 25972889 PMC4413779

[B27] Hazen S. P. Schultz T. F. Pruneda-Paz J. L. Borevitz J. O. Ecker J. R. Kay S. A. (2005). *LUX ARRHYTHMO* encodes a Myb domain protein essential for circadian rhythms. Proc. Natl. Acad. Sci. U.S.A. 102, 10387–10392. 10.1073/pnas.0503029102 16006522 PMC1177380

[B28] Helfer A. Nusinow D. A. Chow B. Y. Gehrke A. R. Bulyk M. L. Kay S. A. (2011). *LUX ARRHYTHMO* encodes a nighttime repressor of circadian gene expression in the Arabidopsis core clock. Curr. Biol. 21, 126–133. 10.1016/j.cub.2010.12.021 21236673 PMC3057456

[B29] Hermans C. Vuylsteke M. Coppens F. Craciun A. Inzé D. Verbruggen N. (2010a). Early transcriptomic changes induced by magnesium deficiency in *Arabidopsis thaliana* reveal the alteration of circadian clock gene expression in roots and the triggering of abscisic acid-responsive genes. New Phytol. 187, 119–131. 10.1111/j.1469-8137.2010.03258.x 20406411

[B30] Hermans C. Vuylsteke M. Coppens F. Cristescu S. M. Harren F. J. M. Inzé D.. (2010b). Systems analysis of the responses to long-term magnesium deficiency and restoration in *Arabidopsis thaliana*. New Phytol. 187, 132–144. 10.1111/j.1469-8137.2010.03257.x 20412444

[B31] Hicks K. A. Albertson T. M. Wagner D. R. (2001). *EARLY FLOWERING*3 encodes a novel protein that regulates circadian clock function and flowering in Arabidopsis. Plant Cell 13, 1281–1292. 10.1105/tpc.13.6.1281 11402160 PMC135582

[B32] Hsu P. Y. Devisetty U. K. Harmer S. L. (2013). Accurate timekeeping is controlled by a cycling activator in Arabidopsis. eLife 2:e00473. 10.7554/eLife.00473 23638299 PMC3639509

[B33] Huang W. Pérez-García P. Pokhilko A. Millar A. J. Antoshechkin I. Riechmann J. L.. (2012). Mapping the core of the Arabidopsis circadian clock defines the network structure of the oscillator. Science 336, 75–79. 10.1126/science.1219075 22403178

[B34] Imaizumi T. Schultz T. F. Harmon F. G. Ho L. A. Kay S. A. (2005). FKF1 F-Box protein mediates cyclic degradation of a repressor of CONSTANS in Arabidopsis. Science 309, 293–297. 10.1126/science.1110586 16002617

[B35] Ito S. Nakamichi N. Kiba T. Yamashino T. Mizuno T. (2007). Rhythmic and light-inducible appearance of clock-associated pseudo-response regulator protein PRR9 through programmed degradation in the dark in *Arabidopsis thaliana*. Plant Cell Physiol. 48, 1644–1651. 10.1093/pcp/pcm122 17890242

[B36] Ito S. Niwa Y. Nakamichi N. Kawamura H. Yamashino T. Mizuno T. (2008). Insight into missing genetic links between two evening-expressed pseudo-response regulator genes TOC1 and PRR5 in the circadian clock-controlled circuitry in *Arabidopsis thaliana*. Plant Cell Physiol. 49, 201–213. 10.1093/pcp/pcm178 18178585

[B37] Johansson M. Staiger D. (2015). Time to flower: interplay between photoperiod and the circadian clock. J. Exp. Bot. 66, 719–730. 10.1093/jxb/eru441 25371508

[B38] Kawamura M. Ito S. Nakamichi N. Yamashino T. Mizuno T. (2008). The Function of the clock-associated transcriptional regulator CCA1 (CIRCADIAN CLOCK-ASSOCIATED 1) in *Arabidopsis thaliana*. Biosci. Biotechnol. Biochem. 72, 1307–1316. 10.1271/bbb.70804 18460819

[B39] Khanna R. Shen Y. Toledo-Ortiz G. Kikis E. A. Johannesson H. Hwang Y.-S.. (2006). Functional profiling reveals that only a small number of phytochrome-regulated early-response genes in Arabidopsis are necessary for optimal deetiolation. Plant Cell 18, 2157–2171. 10.1105/tpc.106.042200 16891401 PMC1560915

[B40] Kiba T. Henriques R. Sakakibara H. Chua N.-H. (2007). Targeted degradation of PSEUDO-RESPONSE REGULATOR5 by an SCFZTL complex regulates clock function and photomorphogenesis in *Arabidopsis thaliana*. Plant Cell 19, 2516–2530. 10.1105/tpc.107.053033 17693530 PMC2002626

[B41] Kikis E. A. Khanna R. Quail P. H. (2005). *ELF4* is a phytochrome-regulated component of a negative-feedback loop involving the central oscillator components CCA1 and LHY. Plant J. 44, 300–313. 10.1111/j.1365-313X.2005.02531.x 16212608

[B42] Kim J.-Y. Song H.-R. Taylor B. L. Carré I. A. (2003). Light-regulated translation mediates gated induction of the Arabidopsis clock protein LHY. EMBO J. 22, 935–944. 10.1093/emboj/cdg075 12574129 PMC145435

[B43] Kim T.-S. Kim W. Y. Fujiwara S. Kim J. Cha J.-Y. Park J. H.. (2011). HSP90 functions in the circadian clock through stabilization of the client F-box protein ZEITLUPE. Proc. Natl. Acad. Sci. U.S.A. 108, 16843–16848. 10.1073/pnas.1110406108 21949396 PMC3189077

[B44] Kim W.-Y. Fujiwara S. Suh S.-S. Kim J. Kim Y. Han L.. (2007). ZEITLUPE is a circadian photoreceptor stabilized by GIGANTEA in blue light. Nature 449, 356–360. 10.1038/nature06132 17704763

[B45] Kolmos E. Herrero E. Bujdoso N. Millar A. J. Tóth R. Gyula P.. (2011). A reduced-function allele reveals that EARLY FLOWERING3 repressive action on the circadian clock is modulated by phytochrome signals in Arabidopsis. Plant Cell 23, 3230–3246. 10.1105/tpc.111.088195 21908721 PMC3203447

[B46] Li G. Siddiqui H. Teng Y. Lin R. Wan X.-Y. Li J.. (2011). Coordinated transcriptional regulation underlying the circadian clock in Arabidopsis. Nat. Cell Biol. 13, 616–622. 10.1038/ncb2219 21499259

[B47] Locke J. C. Millar A. J. Turner M. S. (2005a). Modelling genetic networks with noisy and varied experimental data: the circadian clock in *Arabidopsis thaliana*. J. Theor. Biol. 234, 383–393. 10.1016/j.jtbi.2004.11.038 15784272

[B48] Locke J. C. W. Kozma-Bognár L. Gould P. D. Fehér B. Kevei E. Nagy F.. (2006). Experimental validation of a predicted feedback loop in the multi-oscillator clock of *Arabidopsis thaliana*. Mol. Syst. Biol. 2:59. 10.1038/msb4100102 17102804 PMC1682024

[B49] Locke J. C. W. Southern M. M. Kozma-Bognár L. Hibberd V. Brown P. E. Turner M. S.. (2005b). Extension of a genetic network model by iterative experimentation and mathematical analysis. Mol. Syst. Biol. 1:2005.0013. 10.1038/msb4100018 16729048 PMC1681447

[B50] Makino S. Kiba T. Imamura A. Hanaki N. Nakamura A. Suzuki T.. (2000). Genes encoding pseudo-response regulators: insight into His-to-Asp phosphorelay and circadian rhythm in *Arabidopsis thaliana*. Plant Cell Physiol. 41, 791–803. 10.1093/pcp/41.6.791 10945350

[B51] Martinez-García J. F. Huq E. Quail P. H. (2000). Direct targeting of light signals to a promoter element-bound transcription factor. Science 288, 859–863. 10.1126/science.288.5467.859 10797009

[B52] Más P. Kim W.-Y. Somers D. E. Kay S. A. (2003). Targeted degradation of TOC1 by ZTL modulates circadian function in *Arabidopsis thaliana*. Nature 426, 567–570. 10.1038/nature02163 14654842

[B53] Matsushika A. Imamura A. Yamashino T. Mizuno T. (2002). Aberrant expression of the light-inducible and circadian-regulated APRR9 gene belonging to the circadian-associated APRR1/TOC1 quintet results in the phenotype of early flowering in *Arabidopsis thaliana*. Plant Cell Physiol. 43, 833–843. 10.1093/pcp/pcf118 12198185

[B54] Matsushika A. Makino S. Kojima M. Mizuno T. (2000). Circadian waves of expression of the APRR1/TOC1 family of pseudo-response regulators in *Arabidopsis thaliana*: insight into the plant circadian clock. Plant Cell Physiol. 41, 1002–1012. 10.1093/pcp/pcd043 11100772

[B55] Matsushika A. Murakami M. Ito S. Nakamichi N. Yamashino T. Mizuno T. (2007). Characterization of circadian-associated pseudo-response regulators: I. Comparative studies on a series of transgenic lines misexpressing five distinctive PRR genes in *Arabidopsis thaliana*. Biosci. Biotechnol. Biochem. 71, 527–534. 10.1271/bbb.60583 17284849

[B56] McWatters H. G. Kolmos E. Hall A. Doyle M. R. Amasino R. M. Gyula P.. (2007). *ELF4* is required for oscillatory properties of the circadian clock. Plant Physiol. 144, 391–401. 10.1104/pp.107.096206 17384164 PMC1913775

[B57] Mizoguchi T. Wheatley K. Hanzawa Y. Wright L. Mizoguchi M. Song H.-R.. (2002). LHY and CCA1 are partially redundant genes required to maintain circadian rhythms in Arabidopsis. Dev. Cell 2, 629–641. 10.1016/S1534-5807(02)00170-3 12015970

[B58] Mizuno T. Kitayama M. Oka H. Tsubouchi M. Takayama C. Nomoto Y.. (2014a). The EC night-time repressor plays a crucial role in modulating circadian clock transcriptional circuitry by conservatively double-checking both warm-night and night-time-light signals in a synergistic manner in *Arabidopsis thaliana*. Plant Cell Physiol. 55, 2139–2151. 10.1093/pcp/pcu144 25332490

[B59] Mizuno T. Nomoto Y. Oka H. Kitayama M. Takeuchi A. Tsubouchi M.. (2014b). Ambient temperature signal feeds into the circadian clock transcriptional circuitry through the EC night-time repressor in *Arabidopsis thaliana*. Plant Cell Physiol. 55, 958–976. 10.1093/pcp/pcu030 24500967

[B60] Mockler T. C. Michael T. P. Priest H. D. Shen R. Sullivan C. M. Givan S. A.. (2007). The Diurnal project: diurnal and circadian expression profiling, model-based pattern matching, and promoter analysis. Cold Spring Harb. Symp. Quant. Biol. 72, 353–363. 10.1101/sqb.2007.72.006 18419293

[B61] Nagel D. H. Kay S. A. (2012). Complexity in the wiring and regulation of plant circadian networks. Curr. Biol. 22, R648–R657. 10.1016/j.cub.2012.07.025 22917516 PMC3427731

[B62] Nakamichi N. Kiba T. Henriques R. Mizuno T. Chua N.-H. Sakakibara H. (2010). PSEUDO-RESPONSE REGULATORS 9, 7, and 5 are transcriptional repressors in the Arabidopsis circadian clock. Plant Cell 22, 594–605. 10.1105/tpc.109.072892 20233950 PMC2861452

[B63] Nakamichi N. Kita M. Ito S. Yamashino T. Mizuno T. (2005). PSEUDO-RESPONSE REGULATORS, PRR9, PRR7 and PRR5, together play essential roles close to the circadian clock of *Arabidopsis thaliana*. Plant Cell Physiol. 46, 686–698. 10.1093/pcp/pci086 15767265

[B64] Niwa Y. Yamashino T. Mizuno T. (2009). The circadian clock regulates the photoperiodic response of hypocotyl elongation through a coincidence mechanism in *Arabidopsis thaliana*. Plant Cell Physiol. 50, 838–854. 10.1093/pcp/pcp028 19233867

[B65] Nozue K. Covington M. F. Duek P. D. Lorrain S. Fankhauser C. Harmer S. L.. (2007). Rhythmic growth explained by coincidence between internal and external cues. Nature 448, 358–361. 10.1038/nature05946 17589502

[B66] Nusinow D. A. Helfer A. Hamilton E. E. King J. J. Imaizumi T. Schultz T. F.. (2011). The ELF4-ELF3-LUX complex links the circadian clock to diurnal control of hypocotyl growth. Nature 475, 398–402. 10.1038/nature10182 21753751 PMC3155984

[B67] Ohara T. Fukuda H. Tokuda I. T. (2015). An extended mathematical model for reproducing the phase response of *Arabidopsis thaliana* under various light conditions. J. Theor. Biol. 382, 337–344. 10.1016/j.jtbi.2015.07.016 26231414

[B68] Onai K. Ishiura M. (2005). PHYTOCLOCK 1 encoding a novel GARP protein essential for the Arabidopsis circadian clock. Genes Cells 10, 963–972. 10.1111/j.1365-2443.2005.00892.x 16164597

[B69] Para A. Farré E. M. Imaizumi T. Pruneda-Paz J. L. Harmon F. G. Kay S. A. (2007). PRR3 Is a vascular regulator of TOC1 stability in the Arabidopsis circadian clock. Plant Cell 19, 3462–3473. 10.1105/tpc.107.054775 18055606 PMC2174887

[B70] Pokhilko A. Fernández A. P. N. Edwards K. D. Southern M. M. Halliday K. J. Millar A. J. (2012). The clock gene circuit in Arabidopsis includes a repressilator with additional feedback loops. Mol. Syst. Biol. 8:574. 10.1038/msb.2012.6 22395476 PMC3321525

[B71] Pokhilko A. Hodge S. K. Stratford K. Knox K. Edwards K. D. Thomson A. W.. (2010). Data assimilation constrains new connections and components in a complex, eukaryotic circadian clock model. Mol. Syst. Biol. 6:416. 10.1038/msb.2010.69 20865009 PMC2964123

[B72] Pokhilko A. Mas P. Millar A. J. (2013). Modelling the widespread effects of TOC1 signalling on the plant circadian clock and its outputs. BMC Syst. Biol. 7:23. 10.1186/1752-0509-7-23 23506153 PMC3614443

[B73] Rawat R. Takahashi N. Hsu P. Y. Jones M. A. Schwartz J. Salemi M. R.. (2011). REVEILLE8 and PSEUDO-REPONSE REGULATOR5 form a negative feedback loop within the Arabidopsis circadian clock. PLoS Genet. 7:e1001350. 10.1371/journal.pgen.1001350 21483796 PMC3069099

[B74] Salomé P. A. McClung C. R. (2005). PSEUDO-RESPONSE REGULATOR 7 and 9 are partially redundant genes essential for the temperature responsiveness of the Arabidopsis circadian clock. Plant Cell 17, 791–803. 10.1105/tpc.104.029504 15705949 PMC1069699

[B75] Salomé P. A. Weigel D. McClung C. R. (2010). The role of the Arabidopsis morning loop components CCA1, LHY, PRR7, and PRR9 in temperature compensation. Plant Cell 22, 3650–3661. 10.1105/tpc.110.079087 21098730 PMC3015110

[B76] Schaffer R. Ramsay N. Samach A. Corden S. Putterill J. Carré I. A.. (1998). The late elongated hypocotyl mutation of Arabidopsis disrupts circadian rhythms and the photoperiodic control of flowering. Cell 93, 1219–1229. 10.1016/S0092-8674(00)81465-8 9657154

[B77] Schmal C. Leloup J.-C. Gonze D. (2014). Modeling and simulating the *Arabidopsis thaliana* circadian clock using XPP-AUTO. Methods Mol. Biol. 1158, 337–358. 10.1007/978-1-4939-0700-7_23 24792063

[B78] Schmal C. Myung J. Herzel H. Bordyugov G. (2015). A theoretical study on seasonality. Front. Neurol. 6:94. 10.3389/fneur.2015.00094 25999912 PMC4423511

[B79] Schmal C. Reimann P. Staiger D. (2013). A circadian clock-regulated toggle switch explains AtGRP7 and AtGRP8 oscillations in *Arabidopsis thaliana*. PLoS Comput. Biol. 9:e1002986. 10.1371/journal.pcbi.1002986 23555221 PMC3610657

[B80] Schmidt H. Jirstrand M. (2006). Systems Biology Toolbox for MATLAB: a computational platform for research in systems biology. Bioinformatics 22, 514–515. 10.1093/bioinformatics/bti799 16317076

[B81] Seaton D. D. Smith R. W. Song Y. H. MacGregor D. R. Stewart K. Steel G.. (2015). Linked circadian outputs control elongation growth and flowering in response to photoperiod and temperature. Mol. Syst. Biol. 11:776. 10.15252/msb.20145766 25600997 PMC4332151

[B82] Thommen Q. Pfeuty B. Schatt P. Bijoux A. Bouget F. Y. Lefranc M. (2015). Probing entrainment of *Ostreococcus tauri* circadian clock by green and blue light through a mathematical modeling approach. Front. Genet. 6:65. 10.3389/fgene.2015.00065 25774167 PMC4343026

[B83] Troein C. Corellou F. Dixon L. E. van Ooijen G. O'Neill J. S. Bouget F.-Y.. (2011). Multiple light inputs to a simple clock circuit allow complex biological rhythms. Plant J. 66, 375–385. 21219507 10.1111/j.1365-313X.2011.04489.x PMC3130137

[B84] Wang L. Fujiwara S. Somers D. E. (2010). PRR5 regulates phosphorylation, nuclear import and subnuclear localization of TOC1 in the Arabidopsis circadian clock. EMBO J. 29, 1903–1915. 10.1038/emboj.2010.76 20407420 PMC2885927

[B85] Wang Z. Y. Tobin E. M. (1998). Constitutive expression of the *CIRCADIAN CLOCK ASSOCIATED 1 (CCA1)* gene disrupts circadian rhythms and suppresses its own expression. Cell 93, 1207–1217. 10.1016/S0092-8674(00)81464-6 9657153

[B86] Webb A. A. R. Satake A. (2015). Understanding circadian regulation of carbohydrate metabolism in Arabidopsis using mathematical models. Plant Cell Physiol. 56, 586–593. 10.1093/pcp/pcv033 25745029

[B87] Yazdanbakhsh N. Sulpice R. Graf A. Stitt M. Fisahn J. (2011). Circadian control of root elongation and C partitioning in *Arabidopsis thaliana*. Plant Cell Environ. 34, 877–894. 10.1111/j.1365-3040.2011.02286.x 21332506

[B88] Zeilinger M. N. Farré E. M. Taylor S. R. Kay S. A. Doyle F. J. (2006). A novel computational model of the circadian clock in Arabidopsis that incorporates PRR7 and PRR9. Mol. Syst. Biol. 2:58. 10.1038/msb4100101 17102803 PMC1682023

