# Supplementary material for: A Compact Model for the Complex Plant Circadian Clock
Source: Front Plant Sci. 2016 Feb 5;7:74. doi: 10.3389/fpls.2016.00074 (PMC4742534; doi:10.3389/fpls.2016.00074)
Supplement: Supplementary file 2 [file Presentation1.pdf]

Supplementary Material:
A compact model for the complex plant circadian clock

Joëlle De Caluwé [1], Qiying Xiao [2], Christian Hermans [2], Nathalie Verbruggen [2], Jean-Christophe Leloup [1], and Didier Gonze [1,*]
*Correspondence:
Didier Gonze:
dgonze@ulb.ac.be

1 MODEL EQUATIONS

The time evolution of the mRNA and protein levels of the variables CL (CCA1/LHY), P97 (PRR9/PRR7), EL (ELF4/LUX), P51 (PRR5/TOC1), and of the activity of P (PIF3,PIL1) are governed by the following differential equations:

$$\frac{d[CL]_m}{dt} = (v_1 + v_{1L} * L * [P]) * \frac{1}{1 + (\frac{[P97]_p}{K_1})^2 + (\frac{[P51]_p}{K_2})^2} - (k_{1L} * L + k_{1D} * D) * [CL]_m \tag{1}$$

$$\frac{d[CL]_p}{dt} = (p_1 + p_{1L} * L) * [CL]_m - d_1 * [CL]_p \tag{2}$$

$$\frac{d[P97]_m}{dt} = (v_{2L} * L * [P]_p + v_{2A} + v_{2B} * \frac{[CL]_p^2}{K_3^2 + [CL]_p^2}) * \frac{1}{1 + (\frac{[P51]_p}{K_4})^2 + (\frac{[EL]_p}{K_5})^2} - k_2 * [P97]_m \tag{3}$$

$$\frac{d[P97]_p}{dt} = p_2 * [P97]_m - (d_{2D} * D + d_{2L} * L) * [P97]_p \tag{4}$$

$$\frac{d[P51]_m}{dt} = v_3 * \frac{1}{1 + (\frac{[CL]_p}{K_6})^2 + (\frac{[P51]_p}{K_7})^2} - k_3 * [P51]_m \tag{5}$$

$$\frac{d[P51]_p}{dt} = p_3 * [P51]_m - (d_{3D} * D + d_{3L} * L) * [P51]_p \tag{6}$$

$$\frac{d[EL]_m}{dt} = L * v_4 * \frac{1}{1 + (\frac{[CL]_p}{K_8})^2 + (\frac{[P51]_p}{K_9})^2 + (\frac{[EL]_p}{K_{10}})^2} - k_4 * [EL]_m \tag{7}$$

$$\frac{d[EL]_p}{dt} = p_4 * [EL]_m - (d_{4D} * D + d_{4L} * L) * [EL]_p \tag{8}$$

$$\frac{d[P]}{dt} = 0.3 * (1 - P) * D - [P] * L \tag{9}$$

The parameters L and D represent light and darkness, respectively. Their values are L = 1 and D = 0 during light phases, and L = 0 and D = 1 during dark phases.

Hypocotyl growth is controlled by the core clock via the protein PIF (PIF4/PIF5). The time evolution of the mRNA and protein levels of PIF, and the hypocotyl length are described by the following equations:

$$\frac{d[PIF]_m}{dt} = v_5 * \frac{1}{1 + (\frac{[EL]_p}{K_{11}})^2} - k_5 * [PIF]_m \tag{10}$$

$$\frac{d[PIF]_p}{dt} = p_5 * [PIF]_m - (d_{5D} * D + d_{5L} * L) * [PIF]_p \tag{11}$$

$$\frac{dHYP}{dt} = g_1 + g_2 * \frac{[PIF]_p^2}{K_{12}^2 + [PIF]_p^2} \tag{12}$$

The activations and inhibitions are modeled using Hill terms. The Hill coefficients are set to 2, based on multiple reports of clock genes forming both homodimers and heterodimers, with the evening complex being approximated as a heterodimer of *LUX* and *ELF4* (Pokhilko et al. (2012); Fujiwara et al. (2008); Wang et al. (2010); Henriques and Mas (2013); Lu et al. (2009)). When multiple transcription factors act on the same target, several possible equations can be used to describe the resulting regulation.

Here, we used classical equations for mutually exclusive inhibitors, in which the effect of two inhibitors A and B is given by $\frac{1}{1+(\frac{A}{K_A})^n+(\frac{B}{K_B})^n}$, rather than the non-exclusive form used in previous models, in which A and B combine as $\frac{1}{1+(\frac{A}{K_A})^n} * \frac{1}{1+(\frac{B}{K_B})^n}$.

Both forms were considered during the building of the model, and the results obtained were very similar, as can be seen in Supplementary Figure 1. We verified that the qualitative defects of the clock mutants and the light- and entrainment-related properties of the clock hold true regardless of the form of the equations. Although the distinction between competitive and non-competitive inhibition is important in the case of enzymatic kinetics, the use of the Hill equation in describing transcriptional regulation is empirical rather than based on kinetic laws, making the difference less relevant here.

SBML version

An SBML version of the model is available in the BioModels database (`http://www.ebi.ac.uk/biomodels`) under the identifier number MODEL1601130000.

2 COST FUNCTION

In 8L:16D, the cost function required *CCA1/LHY* mRNA to peak around dawn in the wild type, assigning a score of 0 for a peak between ZT22 and ZT4. This condition was sufficient to ensure the proper phase of all the variables, therefore no additional constraints were added. No conditions were imposed on any of the mutants.

The wild type and all mutants were required to have a detectable rhythm in constant light, defined as all variables having a minimal value of 0.1, as well as a minimal difference of 10% between their minimum and maximum values. The wild type was also required to have a detectable rhythm in continuous darkness. Any solution that did not meet those criteria was considered to be arrhythmic. An arrhythmic wild type incurred a very large score penalty.

The free-running period was calculated by computing the average time between successive maxima of each variable. The wild type obtained a score of 0 for a period between 24 and 25 hours in continuous light, and between 25 and 28 hours in continuous darkness. The *cca1/lhy*, *prr5/toc1*, and weak *elf4/lux* mutants received a zero score if their period in continuous light was at least 1h shorter than the wild type, with no lower bound. The *prr9/prr7* mutant received a zero score if its period was at least 1h longer than the wild type, with no upper bound.

Parameter optimization for the hypocotyl growth module was done independently of the main clock module. Only the wild type was used for this fitting. The system was simulated for 360 hours, then the hypocotyl length was reset to zero and another 120h simulation was perfomed. The *PIF4/5* mRNA level was fitted to experimental data in 8L:16D, 12L:12D, and 16L:8D, obtained from the DIURNAL database. Relative hypocotyl lengths in the WT after five days in photoperiods ranging from 0 to 24h in increments of 3h were fitted to data from Niwa et al. (2009), shown in Figure 9.

3 SUPPLEMENTARY TABLES

Supplementary Table 1. Parameter values for the core clock model.
The units for concentrations are tentatively given as nanomolars. However, the simulated mRNA and protein profiles are always normalized and shown as relative values.

Parameter description	Name	Value	Units
CL synthesis	v_1	4.6	nM.h^{-1}
CL light-induced synthesis	v_{1L}	3.0	nM.h^{-1}
P97 synthesis	v_{2A}	1.3	nM.h^{-1}
P97 CL-induced synthesis	v_{2B}	1.5	nM.h^{-1}
P97 light-induced synthesis	v_{2L}	5.0	nM.h^{-1}
P51 synthesis	v_3	1.0	nM.h^{-1}
EL synthesis	v_4	1.5	nM.h^{-1}
CL mRNA degradation (light)	k_{1L}	0.53	h^{-1}
CL mRNA degradation (dark)	k_{1D}	0.21	h^{-1}
P97 mRNA degradation	k_2	0.35	h^{-1}
P51 mRNA degradation	k_3	0.56	h^{-1}
EL mRNA degradation	k_4	0.57	h^{-1}
CL translation	p_1	0.76	h^{-1}
CL light-induced translation	p_{1L}	0.42	h^{-1}
P97 translation	p_2	1.0	h^{-1}
P51 translation	p_3	0.64	h^{-1}
EL translation	p_4	1.0	h^{-1}
CL degradation	d_1	0.68	h^{-1}
P97 degradation (dark)	d_{2D}	0.50	h^{-1}
P97 degradation (light)	d_{2L}	0.30	h^{-1}
P51 degradation (dark)	d_{3D}	0.48	h^{-1}
P51 degradation (light)	d_{3L}	0.78	h^{-1}
EL degradation (dark)	d_{4D}	1.2	h^{-1}
EL degradation (light)	d_{4L}	0.38	h^{-1}
Inhibition: CL by P97	K_1	0.16	nM
Inhibition: CL by P51	K_2	1.2	nM
Activation: P97 by CL	K_3	0.24	nM
Inhibition: P97 by P51	K_4	0.23	nM
Inhibition: P97 by EL	K_5	0.30	nM
Inhibition: P51 by CL	K_6	0.46	nM
Inhibition: P51 by itself	K_7	2.0	nM
Inhibition: EL by CL	K_8	0.36	nM
Inhibition: EL by P51	K_9	1.9	nM
Inhibition: EL by EL	K_{10}	1.9	nM

It should be noted that the optimization process yielded several sets of parameters with a similar low score, none of which provide a perfect fit for every single aspect of the clock, which is not unexpected given the high degree of simplification of the model. Those parameter sets can be roughly divided into two groups depending on how well they reproduce the *elf4/lux* and *prr5/toc1* mutants. One group correctly predicts the levels of other clock genes and the short period of *prr5/toc1* but has low *PRR9/PRR7* and high *CCA1/LHY* in *elf4/lux* mutants (whereas they should be high and low, respectively). The other group reproduces the clock defects of *elf4/lux* but predicts an almost unaffected *prr5/toc1* mutant. Both are equally successful at modelling the *cca1/lhy* and *prr9/prr7* mutants. The other clock properties presented here (entrainment, photoperiodic adaptation) are independent of the choice of parameter sets. The set presented here was chosen as a compromise between the two extremes.

Supplementary Table 2. Parameter values for the hypocoty growth model.

Parameter description	Name	Value	Units
PIF synthesis	v_5	0.10	nM.h^{-1}
PIF mRNA degradation	k_5	0.14	h^{-1}
PIF translation	p_5	0.62	h^{-1}
PIF protein degradation (light)	d_{5L}	4.0	h^{-1}
PIF protein degradation (dark)	d_{5D}	0.52	h^{-1}
Baseline hypocotyl growth	g_1	0.01	mm.h^{-1}
PIF-induced hypocotyl growth	g_2	0.12	mm.h^{-1}
Inhibition: PIF by EL	K_{11}	0.21	nM
Activation: growth by PIF	K_{12}	0.56	nM

4 SUPPLEMENTARY FIGURES

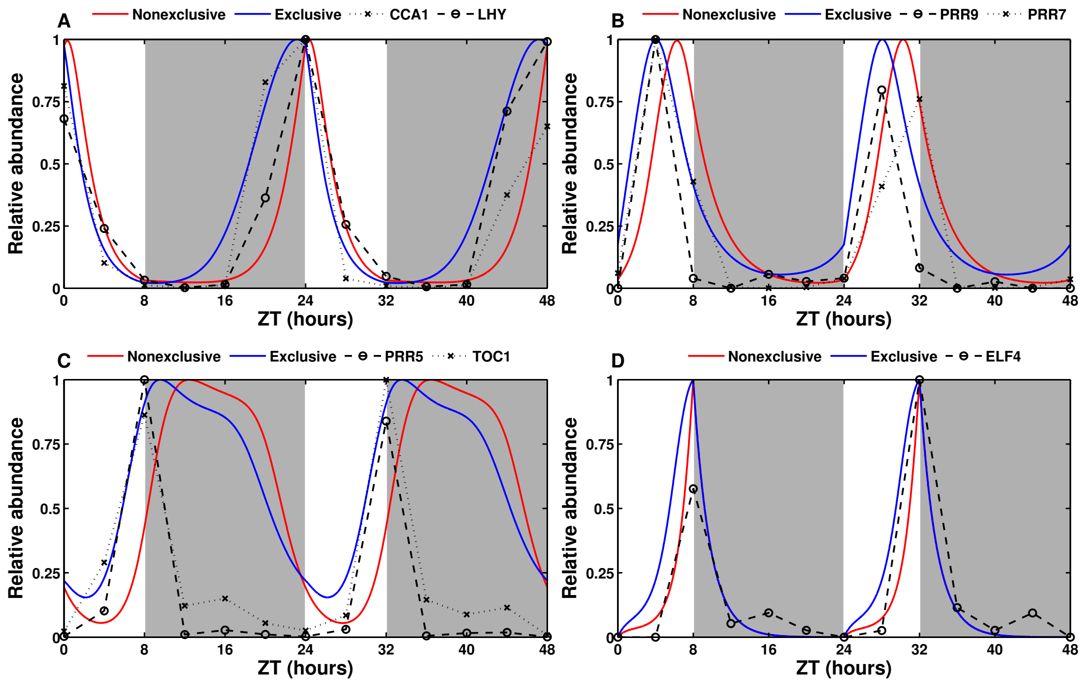

Supplementary Figure 1. Effect of the Hill term on the system.
The experimental data (in black) is the same as presented in Figure 1 of the main text. The blue lines are simulated profiles using the ”exclusive inhibitions” variant of the model, which is the one presented in the main text. The red curves are simulations of a ”nonexclusive inhibitions” variant, using the same parameter values with no further optimization.

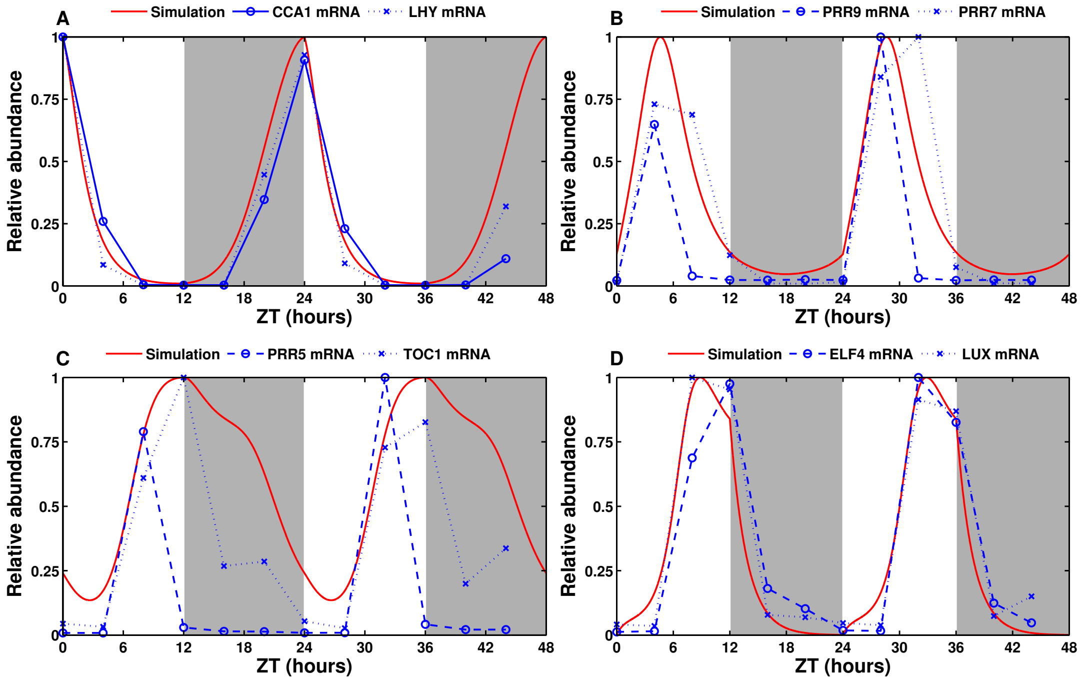

Supplementary Figure 2. Simulated and experimental expression profiles in 12L:12D conditions. **(A)** Simulated and experimentally measured *CCA1* and *LHY* mRNA abundance. **(B)** Simulated and experimentally measured *PRR9* and *PRR7* mRNA abundance. **(C)** Simulated and experimentally measured *PRR5* and *TOC1* mRNA abundance. **(D)** and experimentally measured *ELF4* and *LUX* mRNA abundance. All values are normalized to their respective maximum. The experimental data was obtained from the DIURNAL database. (Mockler et al. (2007))

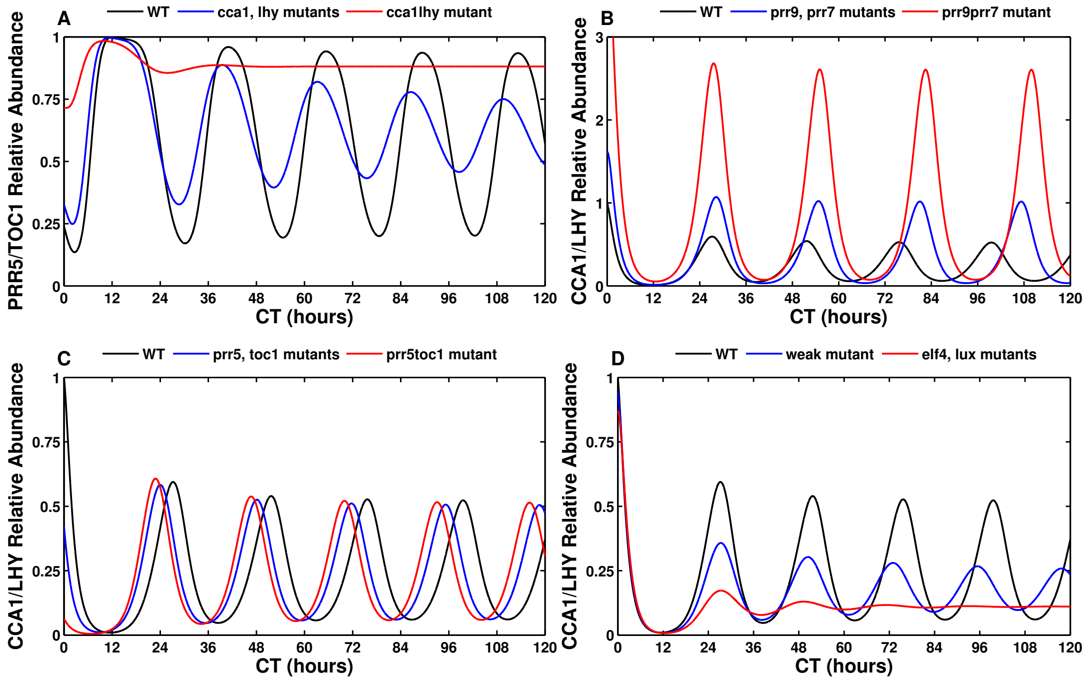

Supplementary Figure 3. Simulated clock mutants in continuous light.
The wild type is in black, single mutants in blue (simulated by dividing the synthesis rate of the affected gene by 2), and the double mutants in red (simulated by dividing the synthesis rate of the affected gene by 10). **(A)** PRR5/TOC1 expression in *cca1, lhy* and *cca1lhy* mutants. The single mutant has a shortened period, while the oscillations in the double mutant quickly damp to undetectable levels. **(B)** CCA1/LHY expression in *prr9, prr7* and *prr9prr7* mutants. Both the single and double mutants have a long period and elevated CCA1/LHY levels. **(C)** CCA1/LHY expression in *prr5, toc1* and *prr5toc1* mutants. The simulated mutants are only slightly affected in continuous light, in contrast to the severely reduced levels of CCA1/LHY obtained in light/dark cycles (see Figure 4 of the main text). **(D)** CCA1/LHY expression in *ec* mutants. These simulated mutants show an opposite effect to the *prr5/toc1* mutants, being almost unaffected in light/dark cycles but severely impaired in continuous light.

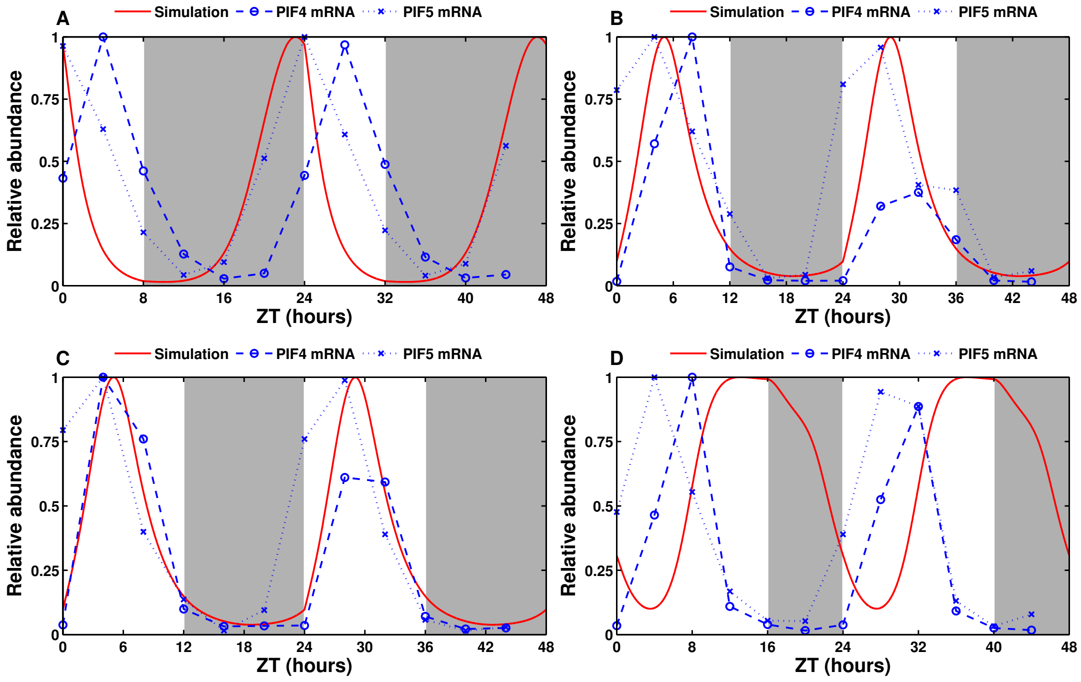

Supplementary Figure 4. Simulated and experimental PIF4 and PIF5 expression profiles in short (8L:16D), even (12L:12D), and long days (16L:8D).
(A) Short days (8L:16D). **(B)** and **(C)** 12L:12D conditions, two different experiments. **(D)** Long days (16L:8D). The data in **(D)** is in the *Ler* background. All data from the DIURNAL database. (Mockler et al. (2007))

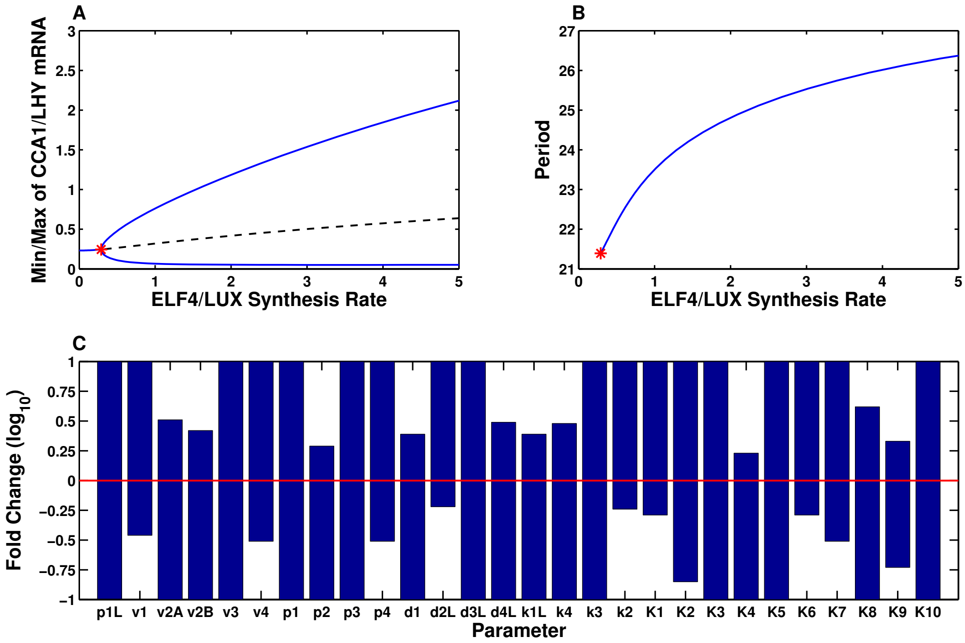

Supplementary Figure 5. Examples of model parameter analysis.
(A) Bifurcation diagram: maximum and minimum values of simulated CCA1/LHY mRNA as a function of ELF4/LUX synthesis rate. The red point is a Hopf bifurcation point. The dashed black line is the unstable steady state. On the left of the bifurcation point, the system displays damped oscillations and converges to a stable steady state. **(B)** Bifurcation diagram: free-running period as a function of ELF4/LUX synthesis rate. **(C)** Sensitivity analysis. The bars represent the range of values for each parameter that produce a detectable free-running rhythm, expressed as a fold change from the optimal value provided in Supplementary Table 1.

REFERENCES

Fujiwara, S., Wang, L., Han, L., Suh, S.-S., Salome, P. A., McClung, C. R., et al. (2008). Post-translational regulation of the Arabidopsis circadian clock through selective proteolysis and phosphorylation of pseudo-response regulator proteins. *Journal of Biological Chemistry* 283, 23073–23083. doi:10.1074/jbc.M803471200

Henriques, R. and Mas, P. (2013). Chromatin remodeling and alternative splicing: pre- and post-transcriptional regulation of the Arabidopsis circadian clock. *Seminars in Cell & Developmental Biology* 24, 399–406. doi:10.1016/j.semcdb.2013.02.009

Lu, S. X., Knowles, S. M., Andronis, C., Ong, M. S., and Tobin, E. M. (2009). CIRCADIAN CLOCK ASSOCIATED1 and LATE ELONGATED HYPOCOTYL function synergistically in the circadian clock of Arabidopsis. *Plant Physiology* 150, 834–843. doi:10.1104/pp.108.133272

Mockler, T. C., Michael, T. P., Priest, H. D., Shen, R., Sullivan, C. M., Givan, S. A., et al. (2007). The Diurnal project: diurnal and circadian expression profiling, model-based pattern matching, and promoter analysis. *Cold Spring Harbor Symposia on Quantitative Biology* 72, 353–363. doi:10.1101/sqb.2007.72.006

Niwa, Y., Yamashino, T., and Mizuno, T. (2009). The circadian clock regulates the photoperiodic response of hypocotyl elongation through a coincidence mechanism in Arabidopsis thaliana. *Plant & Cell Physiology* 50, 838–854. doi:10.1093/pcp/pcp028

Pokhilko, A., Fernández, A. P. n., Edwards, K. D., Southern, M. M., Halliday, K. J., and Millar, A. J. (2012). The clock gene circuit in Arabidopsis includes a repressilator with additional feedback loops. *Molecular Systems Biology* 8:574. doi:10.1038/msb.2012.6

Wang, L., Fujiwara, S., and Somers, D. E. (2010). PRR5 regulates phosphorylation, nuclear import and subnuclear localization of TOC1 in the Arabidopsis circadian clock. *The EMBO Journal* 29, 1903–1915. doi:10.1038/emboj.2010.76
